# Surface Plasmon Resonance-Based Fiber Optic Sensors Utilizing Molecular Imprinting

**DOI:** 10.3390/s16091381

**Published:** 2016-08-29

**Authors:** Banshi D. Gupta, Anand M. Shrivastav, Sruthi P. Usha

**Affiliations:** Physics Department, Indian Institute of Technology Delhi, New Delhi 110016, India; anand.mhn734@gmail.com (A.M.S.); sruthiprasoodu59@gmail.com (S.P.U.)

**Keywords:** optical fiber, sensor, surface plasmon resonance, molecular imprinting

## Abstract

Molecular imprinting is earning worldwide attention from researchers in the field of sensing and diagnostic applications, due to its properties of inevitable specific affinity for the template molecule. The fabrication of complementary template imprints allows this technique to achieve high selectivity for the analyte to be sensed. Sensors incorporating this technique along with surface plasmon or localized surface plasmon resonance (SPR/LSPR) provide highly sensitive real time detection with quick response times. Unfolding these techniques with optical fiber provide the additional advantages of miniaturized probes with ease of handling, online monitoring and remote sensing. In this review a summary of optical fiber sensors using the combined approaches of molecularly imprinted polymer (MIP) and the SPR/LSPR technique is discussed. An overview of the fundamentals of SPR/LSPR implementation on optical fiber is provided. The review also covers the molecular imprinting technology (MIT) with its elementary study, synthesis procedures and its applications for chemical and biological anlayte detection with different sensing methods. In conclusion, we explore the advantages, challenges and the future perspectives of developing highly sensitive and selective methods for the detection of analytes utilizing MIT with the SPR/LSPR phenomenon on optical fiber platforms.

## 1. Introduction

During the last three decades, a tremendous amount of work has been carried out on fiber optic sensors (FOS) for applications in energy, the environment, biomedicine, agriculture, the food industry, buildings and many others due to their unique abilities of supporting biocompatibility, remote sensing and online monitoring with the possibility of miniaturized probes for point of care possibilities [1,2]. The main advantages of using optical fiber for sensing are ease of handling, low weight, low cost, immunity to electromagnetic interference, low power operation, withstanding harsh environment, etc. The optical fiber, in many cases, has replaced traditional substrates such as prisms, electrodes, etc. for the measurement of electrical and mechanical parameters such as strain, stress, magnetic field, current, acceleration, vibration [3], chemical parameters such as pH, glucose, and pesticides, gases, biological samples, refractive index and temperature [4]. In fiber optic sensors, the role of fiber can be either intrinsic or extrinsic, depending on whether the optical fiber is involved in the sensing purpose or not. In the case of extrinsic sensors, the fiber is simply used to guide/carry light from a source to the sensor module whereas in the case of intrinsic sensors, the light guiding properties of the optical fiber are modulated by the sensing medium. The modulation can be in terms of intensity, phase, wavelength, polarization, etc. [5]. For sensing various kinds of spectroscopic techniques/methods such as absorptiometry, refractometry, reflectometry, total internal reflection, interferometry, evanescent field, Doppler effect, Faraday effect, luminescence and surface plasmon resonance (SPR) have all been used.

In recent years the SPR technique has become very popular for sensing of various chemical and biological analytes in addition to refractive index of an aqueous medium. This is due to its label free sensing, fast response and high sensitivity. The SPR-based fiber optic sensors rely on the refractive index variation of the sensing medium. To sense, cladding is removed from a small portion of the fiber and is replaced by a thin film of metal. The sensing medium is kept around the metal layer. The properties of the light transmitting through one end of the fiber are affected by the refractive index variation of the sensing medium. The intensity/spectrum of the output/transmitted light is analyzed to determine the change in the refractive index of the medium. SPR-based sensors have been commercialized due to their fast-label free detection with minimum requirement of analyte medium for sensing. The SPR technique utilizes evanescent waves generated at the core-metal interface to excite surface plasmons at the metal-dielectric (sensing medium) interface. The ability to immobilize bioreceptors easily and with much better stability over metal film facilitates its application in the biomedical field. The sensitivity of SPR-based fiber optic sensors can be improved by modifications in their geometry, doping in layers and addition of high index layers [6,7,8,9,10]. The performance parameter of the SPR sensor that needs improvement is the selectivity of the probe for a particular analyte, which can be improved by tailoring the SPR mechanism with molecular imprinting.

Molecular imprinting technology (MIT) is the technique which is used for the construction of template-specific recognition sites in a polymerized medium. In this process the template (an atom, molecule, ion, complex or molecular, macromolecular, or ionic assembly) is frozen in a covalent/non-covalent polymeric phase and the space occupied by the template is made vacant by removing the template with subsequent removal from the polymer [11,12,13]. Thus the vacant space acts as the recognition site towards the template because the vacancy created by the template has the complementary shape and size of the template structure. The polymer thus created is called a molecularly imprinted polymer (MIP). After the innovative work on the synthesis of MIPs using silica matrices by Polyakov in the 1930s [14], MIT has grown to become as a broad area of interest for the scientific community in general. Numerous reviews [15,16,17,18,19,20], articles [21,22,23,24], and books [25,26,27] have been published on the topic of molecular imprinting. This number is increasing continuously which shows the trends and developments in the area of MIP. Sensors based on the molecular imprinting technique are one of the most interesting topics discussed nowadays due to the high specificity and stability of MIPs in harsh environments. Using SPR and fiber optic configuration, the sensing ability of the MIPs is greatly enhanced. In this review we have summarized the combined approaches of the SPR/LSPR phenomenon with molecular imprinting in fiber optic configuration.

In the present article, the research work carried out on optical fiber sensors utilizing SPR and MIT for the detection of various chemical and biological analytes is reviewed. The review, which starts with a discussion on surface plasmon resonance explains the conditions required to bring about the phenomenon, various interrogation schemes to analyse the response in sensing in terms of intensity, phase, polarization and wavelength. Fiber optic-surface plasmon resonance (FO-SPR) implementation is discussed with the parameters which qualify the sensor performance followed by the possibilities of metal nanostructures in place of metallic film to generate localized surface plasmons. Further, the review covers the molecular imprinting technology with a brief introduction, synthesis procedures of molecularly imprinted polymers (MIPs), and the role of each element used for MIP preparation. The sensing mechanism using FO-SPR- and MIP-based sensors and the studies reported on this topic are also discussed in this review. To conclude, the advantages and the future challenges for improving the sensor performance are discussed.

## 2. Surface Plasmon Resonance (SPR)

The phenomenon of SPR has been establishing its potential in its long ambit since 1902, when Wood observed the diffraction of dark and bright bands of light from a grating structure. In 1968, Otto came up with an optical interpretation by considering a setup with an air (dielectric) gap in between prism and metal, where the evanescent wave generated by attenuated total internal reflection at the prism-air interface excites surface plasmons at the air-metal interface. The limitation of the Otto configuration, namely the difficulty in realizing it practically due to the fact the arrangement of air gap thickness needs to be within the penetration depth of the evanescent wave, was overcome by the Kretschmann-Reather configuration in 1968 [28,29,30]. In this configuration, the spacing or gap layer was metal and the evanescent wave generated at the prism-metal interface excites surface plasmons at the metal dielectric interface.

The quantum of charge density oscillations supported by a metal-dielectric interface and propagating along the interface are termed a surface plasmons (SPs) while the associated wave is called as surface plasmon wave (SPW) [31,32,33]. The electric field of a SPW is maximum at the interface and decays exponentially in dielectric as well as metal media. Further, SPW is a TM-polarized wave and hence to excite SPs a p-polarized light is required [34,35]. For excitation, the wave vector of excitation light should match with the wave vector of SPs. From Maxwell’s equations, the general expression for the propagation constant of the surface plasmon wave propagating along the metal-dielectric interface is given by:

(1) $$k_{SP}=\frac{2\pi }{\lambda }\sqrt{\frac{\varepsilon_{m}\varepsilon_{d}}{\varepsilon_{m}+\varepsilon_{d}}}$$

where $k_{SP}$ is the propagation constant of surface plasmon wave, λ is the wavelength of the incident light in free space, $\varepsilon_{m}$ and $\varepsilon_{d}$ are the dielectric constants of metal and dielectric medium which are functions of the wavelength. The metals that are generally used include silver, gold, aluminum and copper at optical wavelengths. The surface plasmon wave has its dependency on the real and imaginary parts of the dielectric constant of the metal thin film and dielectric medium, where the real part regulates the periodic appearance and the imaginary part results in the attenuation of the wave [36].

**Figure 1:**
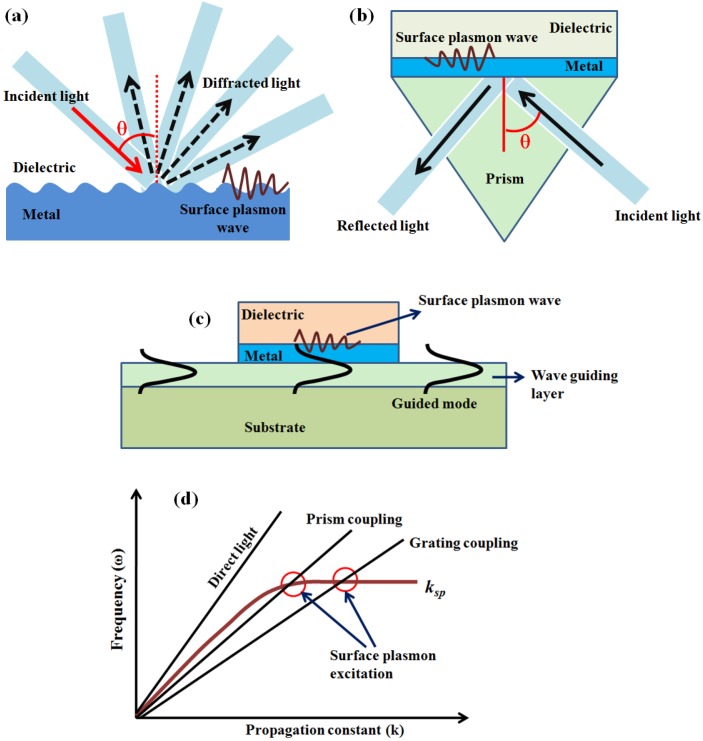
The schematic of (**a**) grating-; (**b**) prism-; (**c**) waveguide-based coupling configurations for SPR generation and (**d**) the surface plasmon excitation possibilities by using evanescent wave in the dispersion curves.

In the case of direct light, SPW holds the momentum/wave vector much higher than that of an incident light and hence cannot excite the SPs. Hence, for the excitation of SPs, the momentum of the excitation light should be increased. Three types of coupling based on gratings, prisms, and waveguides/fibers are used for the excitation of SPs as shown in Figure 1a–c [33]. In grating-based coupling, the diffraction of the light from the grating results in the generation of a diffracted beam in different directions with one of these along the plane of the grating with a wave vector much greater than that of the incident light to match that of the SPs. The period of the grating plays an important role in the excitation of SPs. In the case of prism-based coupling, the attenuated total internal reflection at the prism-metal interface generates an evanescent wave whose propagation constant is given by the following equation:

(2) $$k_{EV}=\frac{2\pi }{\lambda }n_{p}\sin\theta$$

where $n_{p}$ is the refractive index of the material of the prism and *θ* is the angle of incidence of the light. To excite the SPs using a prism-based configuration, the propagation constant of the evanescent wave needs to approach that of the surface plasmons, which idealizes the relation, $k_{EV}=k_{SP}$, which shows the possibility of increasing $k_{EV}$ to match $k_{SP}$ by changing the dielectric constant of the prism. The evanescent wave excites the surface plasmons by its field penetrating through the metal layer. The dispersion curves showing the possibility of excitations by using the configurations compared with the direct light are shown in Figure 1d. For a prism-based coupling, the incoming light incidents at an angle of incidence, $\theta_{res}$, and at the particular angle (greater than critical angle) at which the evanescent wave’s wave vector matches that of the surface plasmon wave, the transfer of energy occurs, the condition being called “resonance”, at which $k_{EV@\theta_{res}}=k_{SP}$. In the case of waveguides, whose structure is similar to that of an optical fiber, the guided modes, which generate evanescent wave to penetrate through the metal layer excite SPs [32,36,37]. The properties of light waves such as phase, polarization, intensity, etc., can be altered by the interaction of surface plasmon waves with the light which in turn changes the propagation constant of the SP.

By using the dielectric medium as the sensing medium the mechanism of SPR can be used for sensing purposes, a first attempt of which was reported in 1983 [37]. For this, the condition for SPR needs the real part of dielectric constant of metal layer to be less than zero, and its absolute value to be greater than that of the prism and its own imaginary part. The analyte to be sensed is kept in contact with the metal layer and any change in its refractive index produces a change in the propagation constant of the surface plasmon wave. Hence, by satisfying the resonance condition, matching of momentum of surface plasmon wave and evanescent wave (due to total internal reflection), a shift in the resonance parameter is observed. The characterization of the sensor depends on the scheme used for the sensing purpose such as angular interrogation, spectral/wavelength interrogation, phase interrogation and intensity interrogation [31,32,36]. In angular interrogation method, a monochromatic light source is used and the angle of incidence of the light is varied to make the propagation constant of the evanescent wave equal to that of the surface plasmon wave. For spectral interrogation, the angle of incidence is kept fixed while the wavelength of the incident light is varied to obtain *λ_res_*. The angle of incidence and wavelength of the incident light are kept fixed to realize the intensity interrogation. In phase interrogation method, the phase difference between p- and s-polarized lights is recorded.

In the case of prism-based coupling discussed above, an angular interrogation scheme is used where at a particular angle of incidence the surface plasmons are excited showing a sudden dip in the light reflectance. The angle corresponding to a sharp dip in reflectance is termed the resonance angle, *θ_res_*. As the refractive index/dielectric constant of the sensing medium changes, the resonance angle changes. This is how the surface plasmon resonance-based sensors work [32]. In the case of systems having sensing layers and analyte, the interaction of analyte to be sensed with the sensing layer changes the propagation constant of the surface plasmon wave which depends on the depth to which the interaction has occurred and the effective refractive index change [36]. This is the principle of sensors based on the SPR technique. The prism-based coupling method is not compatible due to its bulky nature, moving mechanical and optical parts, inability to use it for remote sensing, etc. Hence, researchers started using optical fiber cores instead of prisms, where the evanescent wave can also be produced to excite surface plasmons. The fiber optic SPR sensors are low cost, have miniaturized probes, and are capable of online monitoring and remote sensing with biocompatibility.

## 3. Fiber Optic Surface Plasmon Resonance Sensors

The use of optical fiber for implementing SPR started in 1990 [38,39]. After that the fiber optic SPR (FO-SPR) sensors have been reported for gas, chemistry and biology-related samples [40,41,42,43]. For the fabrication of a FO-SPR sensor, the cladding of the fiber is removed from the fiber structure leaving the bare core exposed for the length of sensing region selected. The metal layer is coated directly on the bare core of the fiber on top of which dielectric and other coatings are used if needed. The length of the sensing region needed is decided in accordance with the metal and dielectric layer chosen for a particular application. The sensing length influences the number of reflections of the ray in the core which further affects the width of the SPR curve [31]. The spectral interrogation scheme is used in fiber, instead of angular interrogation, since a range of guided rays are launched into the fiber [32]. The guided rays generate evanescent waves by total internal reflection which excites surface plasmons at the metal-dielectric interface. The excitation of surface plasmons through the evanescent wave is generally affected by three important factors: the fiber geometry (such as core diameter, probe geometry, V number, sensing length), the incident light properties (like angle of incidence and wavelength) and the metal thin film parameters (choice of metal, film thickness, film roughness) [31,32]. The sensing application involves the calibration curve of analytes by plotting the transmitted power from the fiber as a function of wavelength. The wavelength at which the dip occurs in the SPR curve gives the resonance wavelength, which changes with the change in the refractive index of the sensing medium.

The fiber-based sensor can be used in two configurations namely: transmissive and reflective. In transmissive configuration, the sensing region is fabricated somewhere in the middle of the fiber of chosen length, with a metal coating over its core followed by the dielectric region. The light is coupled from source to the fiber through one end and the reflected light from the interfaces of the sensing region reaching the other end of the fiber is coupled to the analyzer [44]. The reflective configuration involves a thick metal coating on one of the end face of the fiber and the sensing probe is also prepared at this end of the fiber after the removal of cladding. The incident light gets reflected from the mirror-like thick metal coating [40]. Earlier it was reported that implementation of the transmissive concept was difficult with less reproducibility and affecting sensor performance [38], but from the late 90 s till now, the transmissive method-based FO-SPRs have reported high recovery and reproducibility with easy implementation, biocompatibility and best results for a variety of applications.

### 3.1. Performance Parameters

**Figure 2:**
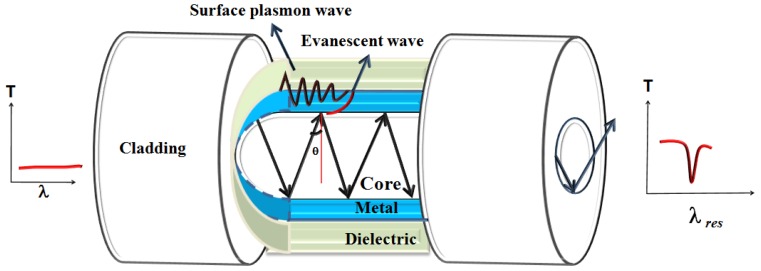
The general optic fiber-based configuration for excitation of SPR.

A general fiber optic SPR sensor configuration is shown in Figure 2 with a coating of metal over the unclad bare core of the optical fiber surrounded by the dielectric sensing medium. The performance of a sensor probe is evaluated in terms of various parameters such as operating range, sensitivity, detection accuracy, selectivity, figure of merit (FOM), regeneration, stability, limit of detection (LOD) and limit of quantification (LOQ).

The operating range of a sensor is an important parameter that decides the application and sustainability of sensor. The structure of the probe also needs to be stable in harsh environments as well as in varying pH and temperature for a particular application. The analysis result of the sensor needs to be stable with time, and should have the ability to recover; otherwise it will be a onetime usable probe. In the case of SPR-based fiber optic sensors, sensitivity (S) is defined as the change in resonance wavelength per unit change in the refractive index of the sensing media. In other words:

(3) $$S=\frac{\delta \lambda_{res}}{\delta n_{d}}$$

where $\lambda_{res}$ is the resonance wavelength at which the evanescent wave’s propagation constant equalizes that of the surface plasmon wave’s and $n_{d}$ is the refractive index of the dielectric sensing medium. The higher the value of S, the better the sensor performance is. Sensitivity can also be calculated by taking the slope of the calibration curve showing the variation of resonance wavelength with analyte concentration or refractive index. The accuracy with which the resonance wavelength can be determined is called detection accuracy. The detection accuracy will be better if the width of the SPR curve is narrower. Hence it is inversely related to the full width at half maximum of the SPR curve. Figure of merit, another important parameter, depends on both full width at half maximum and sensor’s sensitivity [45]. FOM should be maximum for the best performance of a sensor which is usually taken as the ratio of sensitivity to full width at half maximum. The selectivity of the sensor shows how precisely it can detect a particular analyte in the presence of interfering mixtures. Hence in the case of a SPR-based fiber optic sensor with a spectral interrogation method the total shift in resonance wavelength is observed in the presence of interfering mixtures which shows how effectively the sensor can detect the required analyte. The selectivity parameter is important in the case of biological samples and clinical applications. Selectivity is a specific nature of FO-SPR-MIP sensors when compared to all the other existing method-based sensors which will be discussed in the section on molecular imprinting.

Limit of detection (LOD) shows the lowest concentration that can be detected by the sensor depending on its spectral resolution. The factor, spectral resolution (*Δλ*) of the spectrometer, plays an important role since it shows the minimal shift in the resonance wavelength that can be determined and hence decides the lowest concentration detection. Apart from spectral resolution, LOD depends on the sensitivity of the sensor at the near zero concentration of the analyte. It is calculated as the ratio of the spectral resolution of spectrometer to the sensitivity evaluated for the sensor near minimum/zero concentration ($S_{c_{\text{min}}}$) of the analyte. Mathematically, it can be written as:

(4) $$LOD=\frac{\Delta \lambda }{S_{c_{\min}}}$$

Thus, the LOD of the sensor depends on the spectrometer used for the calibration. The possibility of noise in the system and the limitations regarding spectral resolution affect the accuracy of the measurement values [46]. The LOD is an ideal quantity which does not take into account the noise/fluctuation in the measurement. If the fluctuation/noise is greater than the spectral resolution of the spectrometer then a new quantity called limit of quantification (LOQ) is determined which takes into account the standard deviation evaluated for near zero concentration of the analyte. The error/standard deviation in measuring the spectral positions/resonance wavelength quantifies the sensor’s performance. Accordingly, the limit of quantification is calculated as the ratio of the standard deviation in resonance wavelength to the sensor sensitivity near zero concentration, which is different from LOD [47]. Regeneration and stability are the two other parameters which determine a sensor’s performance. The sensor should be recoverable and regenerative to make it cost effective. This can be checked by repeating the measurements on the same sensor probe, several times, in definite periods. The standard deviation determined from repeated measurements shows the recoverable and repeatable nature of the sensor. The stability of the sensor is determined from the fluctuation of the measuring parameter with time after achieving saturation.

### 3.2. Probe Modification

The performance of a fiber optic sensor can be increased by modifying the structural configuration of the fiber. A tapered core configuration of the sensing region can be used instead of the flat model shown in Figure 2, which increases the sensitivity. Here the penetration depth of the evanescent field varies through the sensing region [48,49]. The tapering can be done in a parabolic, linear and exponential-linear way [50]. A high increase in sensitivity has been observed in exponential-linear tapered fiber sensor probes since there would be a decrease in the angle of incidence at the interface which increases the sensitivity. Similarly, the sensitivity also increases with an increase in the tapering ratio [32]. The asymmetric metal coating over a single fiber of uniform waist has been reported to generate multiple resonance dips which boost the dynamic range of sensors [51]. A U-shaped fiber probe profile was reported initially for SPR in 2008 and later the idea was also extended to localized plasmons (Section 4) [52,53]. Sensitivity has been reported to increase with the decrease in the bending radius of fiber up to a certain extent, because further decreases in bending radius cause the angle of incidence to be less than the critical angle, which fails to generate the required condition for SPR. Another configuration is the D-shaped fiber probe which achieves a high resolution and sensitivity due to the multiple peaks/dips [54].

Different designs of SPR-based probes have also been reported using bimetallic coatings which improve the sensitivity, figure of merit and detection accuracy [55,56]. An enhancement in sensitivity of a guided wave SPR sensor has been reported in 2008 by using a dielectric layer having a higher real part over the metal layer coated over a core of optical fiber [57,58]. Thus using different combinations of metals, high index layers, structural configurations and fiber profiles, a great enhancement in fiber optic sensor probe performance can be achieved. In addition to these, the presence of metal nanostructures near the fiber core in the structure of the probe produces localized plasmons instead of the propagating ones, resulting in what termed as localized surface plasmons (LSP), which enhances the sensor performance multiple times.

## 4. Localized Surface Plasmon Resonance

**Figure 3:**
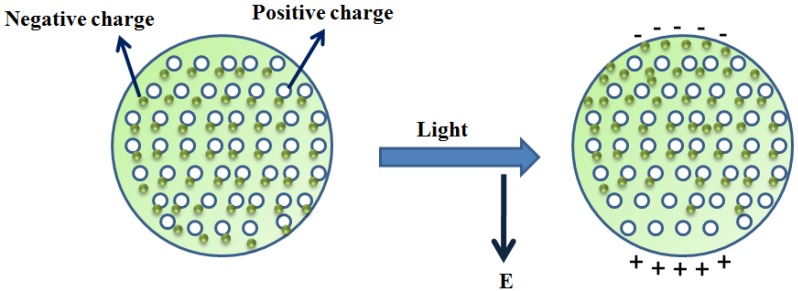
Schematic showing the dipole formation outside the metal nanoparticle in the presence of an oscillating EM field.

The light getting trapped within the metallic nanoparticles having a size smaller than the wavelength of light causes the collective oscillations of conduction electrons, resulting in localized surface plasmons. The displacement of electron clouds with respect to the nuclei occur due to this oscillation which in turn causes attractive and repulsive forces. This allows the transfer of energy to result in strong absorption, scattering and hence enhancement in the EM field. The field due to the resonance inside the nanoparticle creates a dipolar field outside the nanoparticle as shown in Figure 3 [59,60]. The frequency at which resonance occurs is called the resonance frequency. In the case of nanostructures, it is the absorbance that matters. Using the wavelength interrogation method, the change in refractive index can be measured with evaluation of the changing absorbance wavelength [61].

For the implementation of LSPR-based fiber optic sensor, over the unclad bare core of the sensing region, metallic nanoparticles are immobilized. The most used nanoparticles for sensing applications are those of silver and gold because of their sensitivity and biocompatibility. There are several key factors in the nanoparticles/nanostructures which affect the sensor performance. Some of these are the shape and size of the nanostructures, the alignment and separation between nanoparticles and thickness of the nanostructure layer. The shape of the nanoparticle/nanostructure is an important parameter which decides the aspect ratio. Aspect ratio, which is the surface to volume ratio of a nanostructure helps in tuning the sensor’s performance and application. The higher the aspect ratio, the more the absorption is. The sensitivity can be improved by increasing the number of nanoparticles and their size, while limiting the distance between them [62]. The thickness of the nanostructure film also plays a significant role in sensor performance as it decides the interaction of the evanescent field with the sensing medium [63]. The field created by a nanoparticle at its space is affected by the surrounding nanoparticles and it will be different for each nanoparticle. Further, in the case of a single metal nanoparticle, there will be a fast decay in the field with distance from it [64] because the metal nanoparticle acts as a dipole, and for a dipole the electric field decay is inversely proportional to the square of the distance from the dipole [64]. Considering a nanoparticle, the ability of the particle to absorb light which transfers electrons from the ground to an excited state is called absorption cross section while the efficiency of a particle to scatter the photons approaching it is called scattering cross section. The combination of these is termed as extinction cross section which adds both the absorption and scattering efficiency of the particle. The extinction cross section of nanoparticles has a maximum value of πR^2^. Nanostructures such as nanowires, nanorods, nanospheres which will be having different aspect ratios can be used to exploit the LSPR phenomenon and can be fabricated accordingly for different analytes to give high performance sensors. LSPR-based sensors have been reported to have applications in the field of energy, biomedicine, environment, food safety, water control, pharmaceuticals and agriculture [64]. A combination of SPR and LSPR in designing fiber probes for sensing applications has also been reported which greatly enhances the field to improve the sensitivity and limit of detection of the sensor [65,66].

## 5. Molecular Imprinting

As discussed in the Introduction section, the molecular imprinting technique has grown as an attractive area of research due to its unique advantages of “structure predictability, recognition specificity and application universality” [67]. Hence, in this section, we shall focus on the basics of the molecular imprinting technique, synthesis procedures, the role of each element and its applications in sensing. First, we start with the fundamentals of molecular imprinting.

### 5.1. MIP Fundamentals

The MIP synthesis procedure demands the polymerization of functional monomer and the cross-linker around a template [68]. In the first step, template-monomer complexes are prepared with the reaction between a specific template and corresponding required monomer. This step is followed by the cross-linking polymerization reaction around the template-monomer complex. After polymerization, the template is unbound/removed from the achieved complex polymer with the help of the required remover. It may be noted that after the extraction of template molecules from the polymer, the template leaves its complementary imprint in the polymer. These imprints are usually known as the imprinted sites of the template and they contains a three-dimensional network with complementary shape and size to the template, which make the MIPs highly selective for the template molecule.

**Figure 4:**
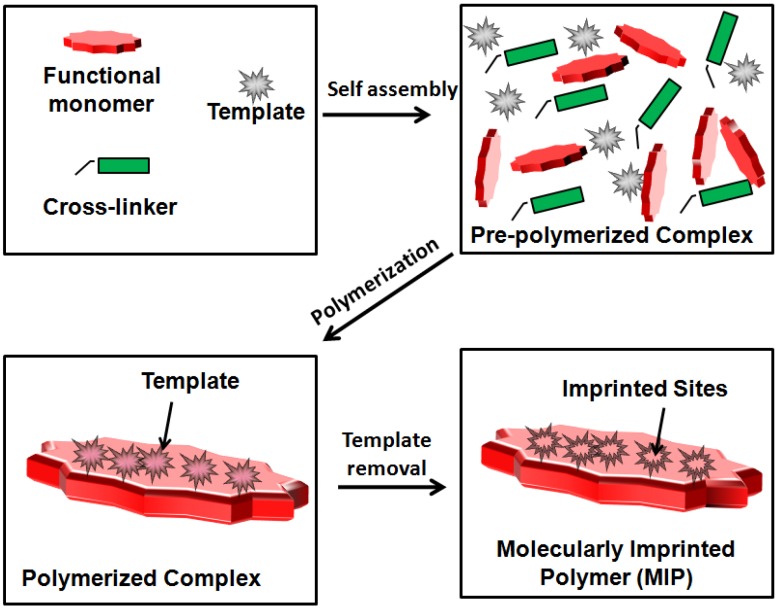
General mechanism of molecular imprinting technique for creating recognition sites for specific template realization.

According to the synthesis procedure and the interaction between template and the functional monomer, molecular imprinting is characterized in two different categories: covalent imprinting and non-covalent imprinting. The first approach of molecular imprinting was developed by Wulff and co-workers [69]. In this process of molecular imprinting, a pre-polymerized compound is prepared between the template and functional monomer by a covalent interaction which is followed by polymerization. The covalent interaction usually occurs between pairs like diol:ketone, amine:aldehyde and acid:amine. Thus, the imprinting of ketals/acetals [70], boronate esters [71] and Schiff’s bases [72] follow the covalent approach. As the interaction between template and functional monomers is of a covalent nature, the porogen (solvent) may be polar [73] for the polymerization process. However, the strong covalent approach results in the slow binding and dissociation of the template [13] which limits the analytical applications of these MIPs. Moreover, this approach is limited due to its template:functional monomers specificity. Mosbach and co-workers pioneered the non-covalent approach for the synthesis of MIPs around the 1990s [74,75]. The method can be processed by non-covalent interactions such as hydrogen bonding, ionic interactions, π-π interactions and Van der Walls forces between the template and functional monomer. The most dominant interaction used for molecular imprinting is hydrogen bonding, which usually occurs between primary amines and functional monomers of methacrylic acid (MAA) groups in a non-polar solvent [17]. Due to its easy synthesis process and quick removal/rebinding of template, non-covalent molecular imprinting has become the most general and popular method of the preparation of MIPs. However, in case of this imprinting approach, the bonds are less selective, non-directional and heterogeneous [76]. A schematic diagram of the synthesis of MIPs is shown in Figure 4. To combine the stability of the covalent method and the rapid removal/unbind of the non-covalent method, a combined method has been developed named semi-covalent imprinting [15]. In this method, the template:functional monomers interact covalently while the rebinding is due to a non-covalent interaction.

### 5.2. MITs for MIP Synthesis

#### 5.2.1. Conventional Methods

A variety of synthesis procedures for MIP preparation have been reported by a number of research groups [77,78,79,80]. Usually, MIP synthesis is done by two types of processes: free radical polymerization and sol-gel processes. In the first type of polymerization method, “bulk polymerization” is the most commonly used procedure for MIP preparation. Small particles of MIPs (up to millimeter size) are prepared by simply crushing or grinding the bulk MIP mechanically. However, in this method, a large amount of template is required and it also suffers from non-uniform distribution of the particle size [81]. Thus, to control the diameter of MIP particles, a few alternative methods are developed such as in-situ (seed) polymerization, suspension polymerization, emulsion polymerization, precipitation polymerization and graft polymerization [79,82]. Among these, suspension polymerization is the simplest method to adopt. The MIPs particles fabricated by this method have sizes in the broad range of 5–50 µm due to the surfactant amount and the stirring speed. However, because of the disturbance of the dispersive medium, the method suffers from the limitation of a wide range of particles size and poor recognition [83]. MIP particles with high yield and mono-dispersity could be synthesized effectively by using the emulsion polymerization method, although, the method cannot be used for biological applications due the presence of the surfactants used [84]. MIP particles with controlled diameter can be achieved by the in-situ polymerization method which is also named seed polymerization. The particles prepared in this method are approximately the same in shape and size which is required for chromatography applications, but the method has its own restrictions of time consumption and use of aqueous suspensions, and it shows poor selectivity [85,86]. More uniform size distribution of particle size could be obtained from the precipitation polymerization method but it requires large amounts of solvent and strictly controlled ambient conditions such as temperature, stirring speed, etc. [87,88]. The sol-gel method is the more advantageous method as compared to the aforementioned methods in terms of its easy synthesis procedure at room temperature, immunity to chemical and thermal influences and the use of eco-friendly porogens such as ethanol, ultrapure water, etc. [12,16].

#### 5.2.2. Surface MIT

This molecular imprinting method was introduced by Mosbach et al. for locating the imprinted sites over a surface by controlling the templates [89]. The method is quite advantageous compared to conventional methods as it provides complete removal of the template and better accessibility to the guest template for recognition. This increases the applications of MIPs for the detection of macromolecules like cells [90], viruses [91] and proteins [92], but the method has the restriction of the limited surface area of the substrate and hence, the finite number of the imprinted sites. These disadvantages could be overcome by applying the method over the substrate having larger surface area.

#### 5.2.3. Nano-MIT

The combined approach of the molecular imprinting and nanotechnology results in the synthesis of nanostructured MIPs [93]. This combinational method greatly enhances the applicability of MIPs because of the improvement in site accessibility and binding capacity of the imprinted materials. N-MIPs in different kinds of nanostructures such as nanoparticles, nanoflowers and nanotubes have been reported by many research groups using different nanofabrication methods [94,95,96]. The abovementioned advantages increase significantly the sensitivity as well as selectivity for broad varieties of analytes.

In the development of molecular imprinting technology, several special imprinting methods were developed. With the need for simultaneous recognition of two templates, multi-template imprinting technology has emerged [97]. In this series several other techniques such as multi-functional monomer imprinting [98], segment imprinting [99], dummy imprinting [100], etc. have been developed. The characterization methods are used for the morphological analysis, monomer template interaction analysis and screening of the monomers of the prepared MIPs. Table 1 shows the various characterization methods and their requirements for the confirmation of MIPs.

### 5.3. Elements of Molecular Imprinting

The synthesis of MIPs generally involves five basic elements: template, functional monomers, cross-linker, initiator and porogen. The type and amount of these elements used for the synthesis of MIPs strongly affect its properties such as specificity, porosity and stability, etc. Among them the “three elements of molecular imprinting” include the template, functional monomers and cross-linkers, to which special attention is paid while investigating MIPs.

#### 5.3.1. Template

The main objective of molecular imprinting is to synthesize polymers having high stability and specificity which are comparable to biological receptors that may be replaced by MIPs in real applications. An ideal template should satisfy three essential conditions: the template should not have functional groups which restrict its polymerization; it should show high chemical stability during the polymerization process and should contain functional groups which can create complexes with functional monomers. Several types of templates such as ions [101,102], organic molecules (pesticides, pharmaceuticals, amino acids, sugars, endocrine disrupting chemicals, etc.) [103,104,105], bio-macromolecules, cells and viruses [90,106] have been reported in MIPs, but the imprinting of proteins and a few bio-macromolecules is still a great challenge for researchers [107].

#### 5.3.2. Functional Monomers

**Figure 5:**
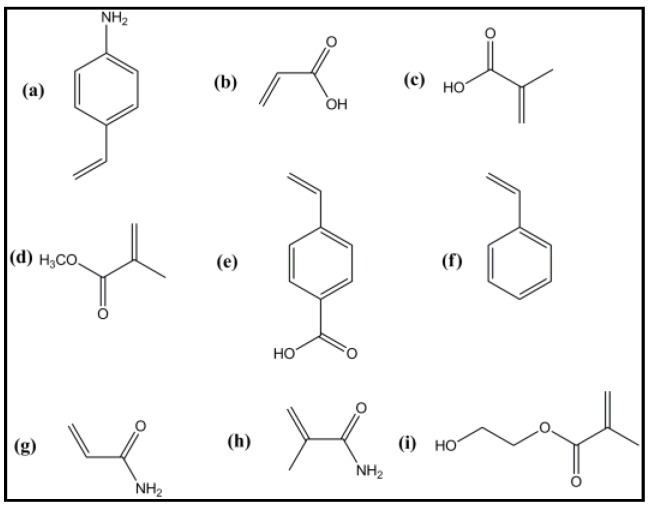
Chemical structures of common functional monomers used for MIP synthesis: (**a**) 4-vinyl aniline; (**b**) acrylic acid (AA); (**c**) methacrylic acid (MAA); (**d**) methyl methacrylate (MMA); (**e**) *p*-vinylbenzoic acid; (**f**) styrene; (**g**) acrylamide (AM); (**h**) methacrylamide; (**i**) 2-hydroxymethacrylate (HEMA).

In MIPs, the functional monomers play an important role in the creation of the pre-polymerization complex with the template that interacts through a specific functional group. Thus, the selection of the necessary functional monomer is quite important. It should be selected such that the template:functional monomers interact strongly and construct specific antigen-antibody or donor-receptor complexes for polymerization. Figure 5 shows some typical functional monomers used for the synthesis of MIPs.

Among these functional monomers, methacrylic acid (MAA) is the “universally” used functional monomer because of its stability for the creation of hydrogen bonds. It is further shown that a high molar fraction of MAA causes a large MIP pore size which plays an important role in the enhancement of the binding capacity of the resulting MIPs [108].

The ratio of functional monomer:template (M/T) is also one of the important parameters which affect the number and stability of MIP binding sites [109,110]. Low M/T ratios lead to the creation of fewer template:monomer complexes due to an insufficient amount of functional monomer. This results in the creation of less imprinted sites and hence poor MIP binding ability, while for high M/T ratios, a limited number of functional monomer binds with the template and the remaining amount of monomer would remain scattered within the MIPs. This results in the non-selective rebinding of the templates with retained monomers, referred as the extreme case of non-imprinted polymer [111].

#### 5.3.3. Cross-Linkers

**Figure 6:**
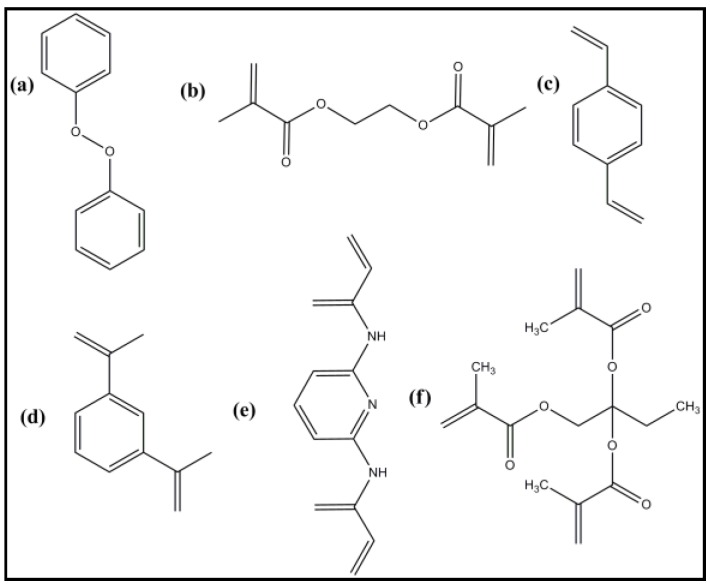
Chemical structures of common cross-linkers for MIP: (**a**) dicumyl peroxide (DCP); (**b**) ethylene glycol dimethacrylate (EGDMA); (**c**) divinylbenzene (DVB); (**d**) 1,3-diisopropenyl benzene; (**e**) 2,6-biscryloylamidopyridine; (**f**) trimethylpropane trimethacrylate (TRIM).

In polymerization process, cross-linkers are used for fixing the functional monomer around the template which leads to the construction of rigid and highly cross-linked MIPs, even after the binding/unbinding of the template. Thus, it can be said that the type and amount of cross-linking agent used for the MIP synthesis strongly affect the rigidity and flexibility of the MIPs. It has been shown by Wulf and Sarhan in 1972 that the degree of cross-linking for the synthesis of MIPs strongly affects the selectivity and binding capability of MIPs [73]. In cases of lower amounts of cross-linker, the stiffness of the MIPs would be poor, which leads to their instability, hence it causes poor selectivity. It was observed that for below 10% of cross-linking, the MIP loses the selective nature [112], while for higher degrees of cross-linking, MIPs show kinetic hindrance towards the reversible binding of the template. This decreases the number of recognition sites per unit mass of MIPs [113]. Thus, to achieve the optimum flexibility/rigidity of the MIPs, the amount and type of cross-linker should be optimized. Figure 6 shows the cross-linkers most commonly used for MIP synthesis.

#### 5.3.4. Porogens (Solvent)

Porogenic solvents are used as a dispersive medium and pore-constructive element for the MIP synthesis process, hence they are essential for the polymerization process. The polarity of the solvent used affects the interaction between template molecule and the functional monomer, thus affecting the adsorption properties of the MIPs. In the case of the non-covalent method, usually non-polar and less polar solvents such as acetonitrile, toluene, chloroform, etc. are used to achieve good imprinting efficiency because the solvent used strongly affects the binding and morphology of the polymer. A theoretical study based on density functional theory (DFT) for the selection of the solvent required for producing MIPs has been reported by Saloni et al. [114]. In this study, the effects of four different solvents: acetone, chloroform, methanol and acetonitrile on the binding energy of template: monomers ratio have been studied. Chloroform is one of the broadly used solvents because it dissolves many of the monomers and templates without suppressing the hydrogen bonding.

#### 5.3.5. Initiators

**Figure 7:**
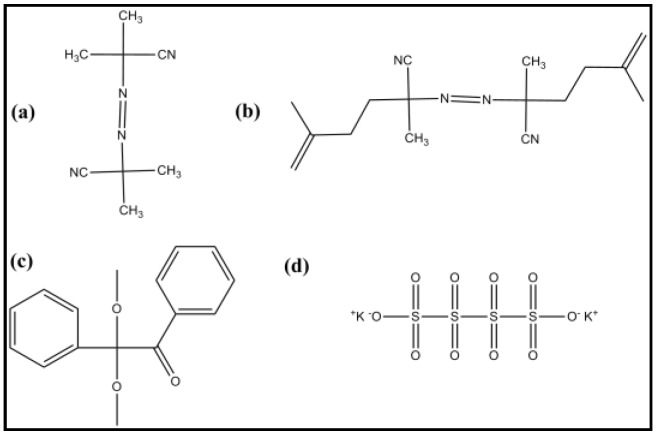
Common initiator structures used in MIP synthesis: (**a**) azobisisobutyronitrile (AIBN); (**b**) 4-4′-azo(4-cyanovaleric acid) (ACID); (**c**) benzoylperoxide (BPO); (**d**) potassium persulfate (KPS).

The majority of MIPs are prepared by free radical polymerization, photopolymerization and electropolymerization processes. Usually compounds with azo- groups, shown in Figure 7, are widely used as initiators [115]. Among these azobisisobutyronitrile (AIBN) is the most commonly used initiator. Prior to polymerization, removal of the oxygen from the pre-polymerized solution is very important, which is performed by bubbling the solution using an inert gas such as argon or nitrogen.

#### 5.3.6. Polymerization Temperature

The equilibrium between template:monomer is achieved by maintaining the surrounding ambient temperature and pressure [116]. It has been reported in earlier studies that for MIPs based on electrostatic interactions, the surrounding temperature should be low because of the strong electrostatic interaction occurring at lower temperatures [117,118]. However, the polymerization process is slow at lower temperature. The pH of the rebinding solvent is also an important parameter which affects the properties of MIPs [119].

### 5.4. Applications of Molecular Imprinting

As discussed in the Introduction section, MIP has high specificity towards the template and it can be applied in harsh conditions like high pH, temperature, etc. Due to its interesting advantageous features, this technique is applied in number of applications such as solid phase extraction (SPE) [120,121,122], chiral discrimination [123], binding assays [124,125,126] and sensing [127,128,129]. Since the present review is based on MIP and fiber optic SPR sensors, here we shall discuss the sensing applications of this combined phenomenon and technology.

#### MIT for Chemical/Biological Sensing

The application of MIPs for the sensing element has been introduced by Mosbach and co-workers for the detection of vitamin K_1_ using a surface imprinted silicon substrate by means of optical detection. After this study, an era of sensing based on MIPs started due to the several beneficial factors like high selectivity and stability, simple and cheap fabrication process and the versatility in many areas such as environmental control, bio-medical diagnostics, drug screening and food analysis. Because of the complementary shape and size of the imprinted sites as that of the template to be detected, the MIPs show high specificity properties which are an essential parameter of a reliable sensing method. This is the reason why a number of sensing schemes based on electrochemical [130,131,132], fluorescence [133,134,135], chemiluminescence [136,137,138], colorometric/UV-Vis spectroscopy [139,140,141], surface plasmon resonance (SPR) [142,143,144], surface enhanced Raman scattering (SERS) [145,146,147] have been published using molecular imprinting technique. Among these, SPR has been developed as a broadly used detection scheme due to its properties of speed, highly sensitive detection and label-free sensing abilities [32,36,39,58]. The SPR sensing fiber optic configuration boosts sensing with factors of simple instrumentation, cost effectiveness, immunity to electromagnetic interference and the ability of performing online and remote sensing applications [1,3,29,50]. Hence, in the next section the sensing mechanism and various sensors reported in the literature using fiber optic SPR and MIT are discussed.

## 6. Fiber Optic SPR Sensor Based on MITs

### 6.1. Sensing Mechanism

As discussed in the earlier section, SPR is sensitive to changes in the dielectric function of the sensing medium. If there exists any change in the dielectric function, then the refractive index of the sensing/recognition layer changes, and a shift in the resonance wavelength would be found using the fiber optic configuration and spectral interrogation method. The shift in resonance wavelength occurs because of the satisfaction of the resonance condition [32,36,148]. In the case of FO-SPR-based MIP sensing, the MIP layer is used as the recognition layer which has the memory imprinted sites of the template molecule to be detected. When the template molecules come near these imprinted sites, they bind non-covalently with the imprinted sites which results in the change in the dielectric function or the effective refractive index of the sensing medium. This has been recognized by the shift in resonance wavelength while recording the transmittance spectra with variation in analyte concentration. The sensitivity of the FO-SPR is further enhanced by the several methods such as including a thin film of material having a high refractive index with nm thickness between the metal layer and the sensing layer [8,57,149] or tapering the fiber core [46,47]. When metal nanostructures are introduced instead of the metal thin film, the LSPR phenomenon occurs and this is more sensitive as compared to SPR studies in the case of biomolecular interactions due to the increased surface area to volume ratio [150]. Although SPR is more sensitive towards the refractive index measurements as compared to LSPR [151], the sensitivity of LSPR in the case of bio-molecular interaction is higher because of confinement of the electric field in a small sensing volume. This causes the LSPR to be more sensitive towards molecular binding, etc. [150]. In the case of LSPR-based sensors, the MIP layer is coated around the metal nanoparticles which work as the sensing medium. Another case of highly selective detection of analytes using the plasmonic phenomenon is the combination approach of localized and propagating SPR phenomena [65,66]. This method shows the highest sensitivity as compared to SPR/LSPR methods.

### 6.2. Experimental Instrumentation

**Figure 8:**
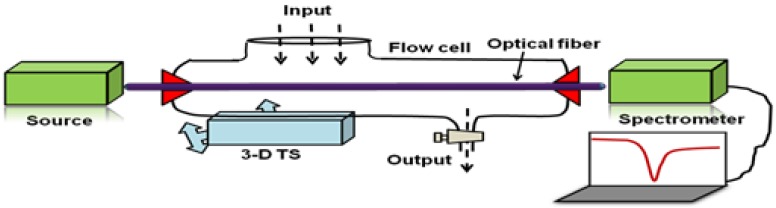
Generic experimental arrangement for fiber optic SPR sensor utilizing MIP as analyte recognition layer.

The typical schematic diagram of the experimental setup used for the characterization of the sensing probe is shown in Figure 8.

Both ends of the fabricated probe are cleaved using a sharp stainless steel blade for the launching of maximum light into the fiber. The probe is fixed in a flow cell with the facilities of inserting and removal of the sample solutions. This is to ensure the interaction of sample solution with the MIP layer/sensing medium. The setup is then mounted on a three-dimensional translational stage to ensure that the maximum light could be guided through the fiber. The light from a polychromatic source is launched through one end of the fiber. This may be referred to as the input end. The light interacts with the sensing medium and changes its characteristics (due to the analyte interaction in MIP layer) which are recorded as the transmitted response at the other end of the fiber using spectrometer which is interfaced with a computer/laptop. In the case of metal nanostructures, usually absorbance is recorded instead of transmittance because of absorption of light by metal nanoparticles. The wavelength corresponding to the resonance condition is called peak absorbance wavelength. In the following section, various sensors reported in the literature for the detection of analytes having applications in environmental monitoring, food/water safety, agricultural production, drug/vitamins detection, etc. are discussed.

### 6.3. Developments on FO-SPR-MIP-Based Sensors

#### 6.3.1. TNT

The explosive detection in aqueous media is quite essential for human health, safety and environmental monitoring reasons. Trinitrotoluene (TNT) is one of the common explosives which are important to detect. Cennamo et al. reported a TNT detection scheme based on the combined approach of FO-SPR and the molecular imprinting technique [152]. SPR was realized by the coating of 60 nm thick gold film over the core of the fiber by a sputtering method. Further, MIP was coated over the Au coated region by spin coating to ensure its uniformity. MIP was prepared using TNT as template, MAA as functional monomer, divinylbenzene (DVB) as cross-linker and AIBN as initiator. The sensing method showed the detection limit of 5.1 × 10^−5^ M while the sensor has the sensitivity of 2.7 × 10^4^ nm/M. The sensitivity of the TNT sensor was improved by incorporating the LSPR phenomenon instead of SPR [153]. LSPR was implemented using five branched Au nanostars which were synthesized by a conventional seed growth method [154]. Au nanostars were dispersed in MIP medium and coated over the core of the fiber. The method of synthesis of the MIP layer was the same as discussed in previous study [152]. After characterization of the sensing probe, the method showed better values of sensitivity and LOD as 8.4 × 10^4^ nm/M and 2.4 × 10^−6^ M, respectively.

#### 6.3.2. Tetracycline (TC)

**Figure 9:**
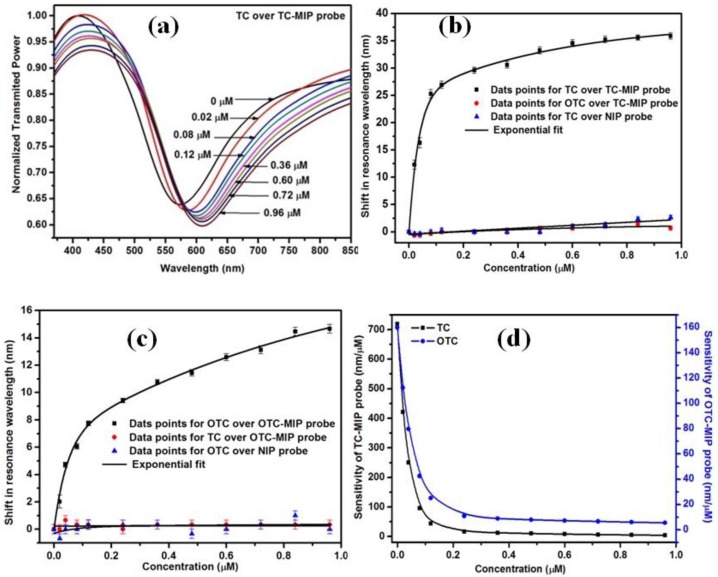
(**a**) SPR response; and shift in resonance wavelength for FO-SPR based (**b**) tetracycline (TC) and (**c**) oxytetracycline (OTC) sensor using molecularly imprinted hydrogel for the concentration range of 0–0.96 µM, and (**d**) the comparison of sensitivities evaluated for TC and OTC imprinted sensors. Reprinted with permission from [157]. Copyright 2013 Royal Society of Chemistry.

Tetracycline is a type of antibiotic which is used for the prevention and treatment of several bacterial infections such as acne, pneumonia, etc. [155,156]. However, large amounts of tetracycline may harm the human body via skin problems, allergic symptoms and skin diseases. Therefore, the monitoring of tetracycline is important to ensure food safety. A TC detection method using MIT and fiber optic SPR technique was reported [157]. The sensing probe was prepared by coating an Ag thin film over the core of the optical fiber followed by a MIP TC layer. MIP TC was synthesized in a polyacrylamide hydrogel medium where acrylamide (AM) and N,N-methylenebisacrylamide (BIS AM) were used as monomer and cross-linking agent while TC was used as template molecule. The sensor operation was checked for the TC concentration range 0–0.96 µM, and for the same concentration change the shift in resonance wavelength was observed to be 35.892 nm. Figure 9a,b show the SPR response and the shift in resonance wavelength for different concentrations of TC sample. Oxytetracycline (OTC) is also a member of the TC family. The detection of OTC was also shown in the same study for which OTC was used as template molecule instead of TC.

The sensing probe was characterized for the OTC concentration range 0–0.96 µM. In this case the shift in resonance wavelength was found to be 14.668 nm with OTC concentration change as shown in Figure 9c. The sensitivity variation of both the probes (MIP-TC and MIP-OTC) is shown in Figure 9d. The response of both the probes for other analyte is negligible as shown in Figure 9b,c.

**Figure 10:**
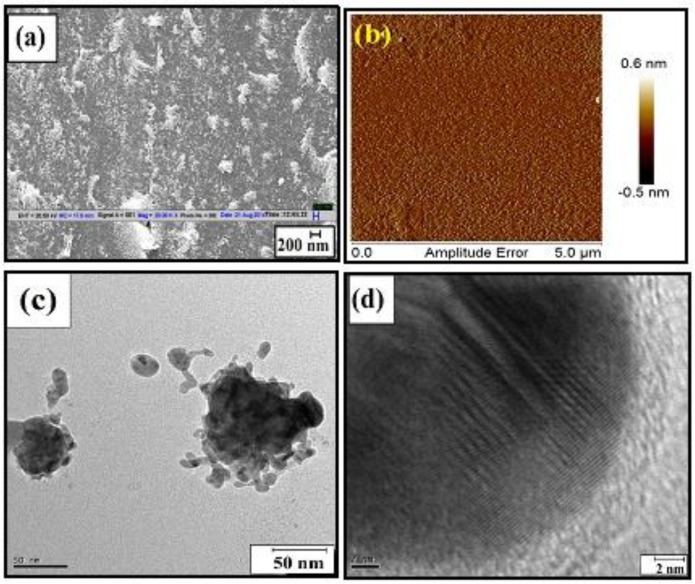
Structural characterization of the silver nanoparticles synthesized in hydrothermal way for the SPR/LSPR based TC sensor with MIP hydrogel. The average size of nanoparticles was found to be in the range of 10–30 nm. Reprinted with permission from [65]. Copyright 2015 Institute of Physics.

The sensitivity of the TC sensor was further improved by Shrivastav et al. by incorporating the combined phenomenon of SPR and LSPR [65]. In this method, the sensor was fabricated by including the Ag nanoparticle layer between Ag and MIP-TC layer. Silver nanoparticles were synthesized hydrothermally [158] and their various characterizations like scanning electron microscopy (SEM), high resolution transmission electron microscopy (HRTEM) and atomic force microscopy (AFM) were reported as shown in Figure 10.

**Figure 11:**
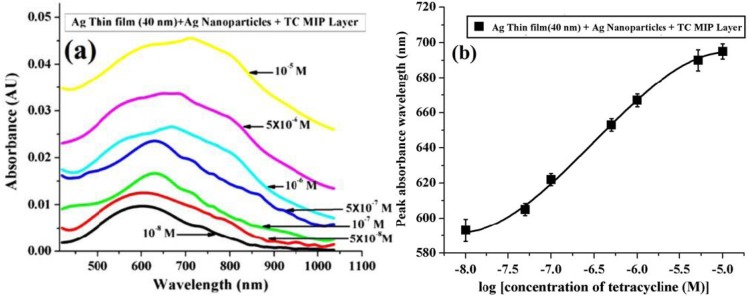
(**a**) The absorbance spectra and (**b**) calibration curve for the SPR/LSPR based TC sensor utilizing MIP hydrogel in the concentration range of 10^−8^–10^−5^ M. Reprinted with permission from [65]. Copyright 2015 Institute of Physics.

The operating range of the sensor was enhanced to 0–10 µM in this work. The shift in peak absorbance wavelength was found to be around 102 nm for the same TC concentration range and shown in Figure 11. The sensing method showed a detection limit of 2.2 × 10^−9^ M for TC.

#### 6.3.3. Vitamin B_3_

**Figure 12:**
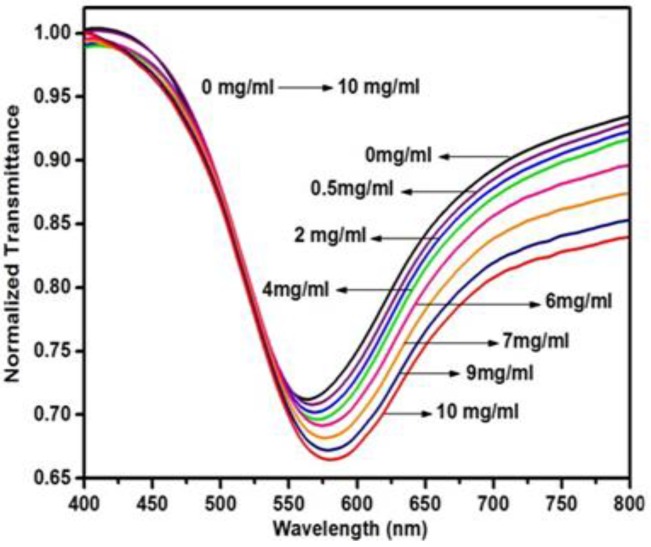
SPR curve recorded for the characterization of vitamin B_3_ sensor using MIP for the concentration range of 0–10 mg/mL. Reprinted with permission from [159]. Copyright 2013 Elsevier.

Among the eight vitamins in the B group necessary for the correct body functioning, B_3_, also known as niacin/3-pyridinecarboxamide, is essential for maintaining a healthy skin, proper breathing and metabolism and to keep the nervous system fully functional. An increase in its amount may create skin problems, diarrhea, wheezing, etc. Hence, the quantification of vitamin B_3_ is necessary in body as well as in pharmaceuticals. In 2013, a molecular imprinted hydrogel-based SPR fiber optic sensor utilizing colloidal crystal templating was reported [159]. The polymer was prepared using vitamin B_3_ with acrylamide/bis acrylamide as cross-linker, TEMED as catalyst and APS as initiator. The fabrication of sensor probe was performed by coating silver film of 40 nm over unclad fiber. The colloidal crystal templates were prepared by dip coating it in diluted solution of monodispersed polysterene spheres. The fiber was then tilted by 15–20° and the polymerized solution was added drop by drop. The fiber was dipped in dimethylbenzene for 30 min to remove the colloidal crystals and then in acetic acid solution for 6 h to create the imprints. The finalized probe was named “colloidal crystal template-vitamin B_3_ imprinted optical fiber probe”. The sensor probe was characterized for analyte concentration of 0 to 10 mg/mL with a red shift in resonance wavelength of 16.651 nm for the SPR curves recorded as shown in Figure 12.

Riboflavin/vitamin B_2_ is another nutrient that belongs to the same family. The sources of riboflavin are egg, milk and leafy vegetables. A riboflavin sensor using SPR and a molecularly imprinted hydrogel has also been reported in the literature [160]. The sensor was characterized for riboflavin concentration range of 0–320 µg/mL where the sensor showed a blue shift in resonance wavelength with the increase in riboflavin concentration.

#### 6.3.4. l-Nicotine

Demands for drug detection methods have been increasing day by day due to their usage in medicines, ready-made foodstuffs and as waste elements in water bodies which affect the human body toxically. One drug, l-nicotine, is an important alkaloid that exists naturally as an organic nitrogen-containing base which affect the body physiologically. Nicotine is reported to affect the nervous system which can result in paralysis and respiratory block. It is actually an addictive, and behaves as a stimulant, which compels the user to use it again. l-Nicotine is a basic material used for medicines in a controlled way and also behaves as a pesticide in a large amount [161]. Hence the analysis of its quantity in medicines and even in tobacco-containing products is important. A fiber optic l-nicotine sensor using SPR and MIP on tapered plastic fiber has been reported [161]. The method was reported to have advantages over the already existing methods for the analysis of nicotine such as chromatography, GC-MS, etc. The ability to fabricate an inexpensive, simple and highly selective sensor using SPR and MIP outperformed all the existing methods and analyses.

The work on nicotine sensing reported by Cennamo et al. used polymeric fibers to exploit their advantages such as flexibility and manipulation possibilities, huge diameter and NA in addition to easy bending of the fibers. Moreover, the plastic material supports smaller bending radii than glass material and is of low cost. To increase the sensitivity, a tapered configuration was applied on the probe. The probe was fabricated on the fiber with core made of PMMA and fluorinated cladding. A sensing region of 10 mm length and taper ratio 1.8 was chosen. Gold film of 60 nm thickness was coated to establish SPR. Over the gold film, a pre-polymerized mix made of MAA as monomer, DVB as cross linker and AIBN as initiator with template-l-nicotine was coated. The molar ratio of template: MAA:DVB was set as 1:4:24 with the mixture properly sonicated and bubbled with nitrogen to remove oxygen. Polymerization was performed at a temperature of 70 °C for 16 h to achieve a thickness of 150 nm. To remove the template molecules, ethanol was used to wash the probe which produced the imprints. The sensor probe was characterized at 25 °C with a halogen lamp and spectrum analyzer having a resolution of 1.5 nm. The characterization was performed by electrochemical method indicating the adsorption of analyte on MIP layer. A right shift in the SPR spectrum was observed with the increase in l-nicotine concentration from 0–10^−2^ M with a linear range up to 10^−3^ M. A sensitivity of 1.3 × 10^4^ nm/M was obtained for probe with taper ratio of 1.8. In addition, the sensor possesses response time of 10 min and LOD of 1.86 × 10^−4^ M.

#### 6.3.5. Melamine

**Figure 13:**
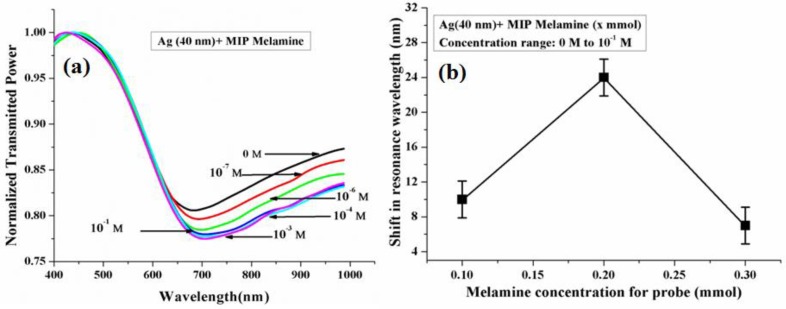
SPR based fiber optic melamine sensor characterized in the range 0–10^−1^ M showing (**a**) SPR response and (**b**) optimization plot for template concentration in MIP. Reprinted with permission from [164]. Copyright 2015 Elsevier.

Melamine is a complex having excess of nitrogen and is extensively used in the manufacturing of plastics, kitchenware, etc. Due to its nitrogen excess, it works as a flame-resistant material [162] and is used to maintain the protein level in milk as an impurity which is calculated by the Kjeldahl method [163]. Its higher concentration in human body creates melamine-cyanurate complexes in the kidneys which are much less soluble in water. Thus, an excess of melamine results in various renal diseases in humans and animals. Further, its excess can cause cancer and damage the reproductive system or result in breathing problems. A fiber optic SPR and molecular imprinting-based melamine detection method was proposed by Shrivastav et al. [164]. For the synthesis of MIP/sensing medium melamine, MAA, ethylene glycol dimethacrylate (EGDMA), AIBN, benzene was used as template, functional monomer, cross-linker, initiator and solvent, respectively. The sensor’s response was checked for a melamine concentration range from 10^−7^ to 10^−1^ M as shown in Figure 13a. The shift in resonance wavelength for this concentration change was found to be of 19.52 nm and the sensor possesses a maximum sensitivity of 10.1 nm/log M near melamine concentration of 10^−7^ M. The melamine (template) concentration was also optimized in this study as shown in Figure 13b as it strongly affects the sensor performance as discussed in an earlier section.

The study was extended by adding a 3 nm thick high index silicon layer between Ag and MIP layer in the probe [165]. Addition of silicon layer improved the sensitivity of the sensor from 10.1 to 76.0 nm/log M; the sensor’s operating range also got increased to 10^−8^–10^−1^ M. The sensing probe showed a better detection limit of 4.3 × 10^−11^ M. This is because of the electric field enhancement at the silicon/sensing medium interface [8,57,58].

#### 6.3.6. Ascorbic Acid

A form of vitamin C, ascorbic acid, is a commonly available nutrient which has anti-oxidizing properties. It is an organic compound that naturally occurs in fruits and vegetables and is available in the form of medicines as well as in some chemically prepared pharmaceutical fluids. An excess intake of it as a food additive results in gastric problems due to its acidic nature. To overcome the limitations such as complexity of the setup, the inability to support online monitoring and remote sensing and needing a pretty long response time for ascorbic acid detection by the existing methods like electrochemical, potentiometry, HPLC, etc., a polyaniline (PANI) MIP-based fiber optic sensor exploiting the principle of SPR was reported [166].

**Figure 14:**
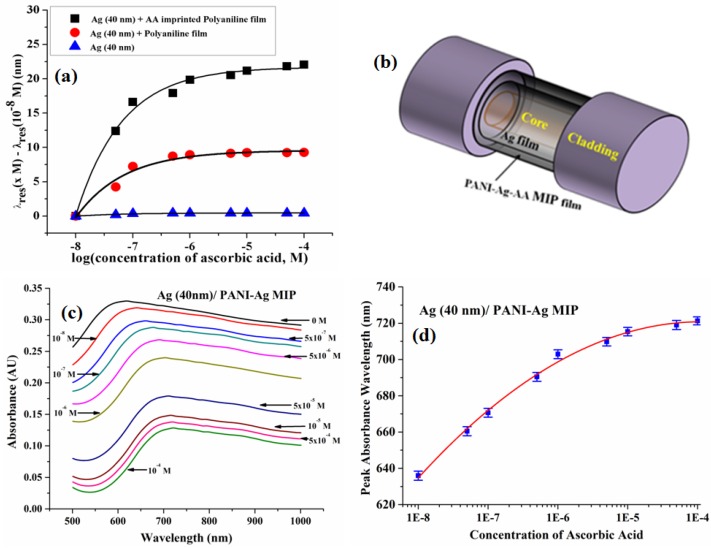
(**a**) Comparison of MIP, NIP and simple Ag film-based SPR sensor in terms of shift in resonance wavelength with varying concentration of ascorbic acid from 10^−8^–10^−4^ M. Reprinted with permission from [166]. Copyright 2015 Springer; (**b**) Schematic diagram of the FO-LSPR + SPR-based PANI-Ag MIP probe; (**c**) the absorbance spectra and (**d**) shift in peak absorbance wavelength for the corresponding probe. Reprinted with permission from [66] Copyright 2016 Institute of Physics.

A synthetic recognition system was fabricated using PANI which involves easy processing to create a mechanically and electrically strong polymer with hydrogen bonds for template binding. The core of the plastic clad silica fiber was used as substrate with 1 cm length of the cladding removed from the central region of the fiber to realize the sensing region. The polymer was prepared using N-methylpyrrolidinone as monomer, ammonium persulphate as oxidizing agent and HCl as solvent. Over the 40 nm thick silver film coated on the unclad core, the prepared polyaniline with template molecule was dip coated and then dipped in water for 5 min to create recognition sites. The characterization of the sensor probe was performed for ascorbic acid concentration range 10^−8^ to 10^−4^ M along with the zero concentration. A total shift of 37 nm in resonance wavelength was reported for ascorbic acid solution having pH 7. A comparison plot for the MIP, NIP and simple Ag film coated probes responses are shown in Figure 14a. A maximum sensitivity of 26.384 × 10^8^ nm/M was evaluated near zero concentration with LOD of 1.28 × 10^−10^ M.

In 2016, an extension of the work was reported employing both SPR + LSPR techniques along with MIP [66]. A nanocomposite of PANI-Ag was prepared by in-situ polymerization with ascorbic acid as template molecule and the ratio of PANI and Ag was optimized for the best probe performance. The probe with 1 cm sensing region and 40 nm thick silver film over unclad core was coated with PANI-Ag-AA nanocomposite (AA-ascorbic acid). The schematic of the SPR+ LSPR probe is shown in Figure 14b. An optimized dipping time of 30 min in nanocomposite and PANI-Ag ratio of 2:5 was obtained from experimental analysis. The optimum removal time of templates from the nanocomposite to create imprinting sites was found to be 1 min. The characterization of the probe was performed for the concentration range of 0 to 10^−8^ M. The absorbance curves for different concentrations are shown in Figure 14c,d with a total shift of 86 nm in peak absorbance wavelength. A linear range was observed for AA concentration range of 0.01–1 μM. A very small response time of 5 s was needed to show the saturation behavior of the curve which surpasses all the existing methods. The sensor possesses 45.1 × 10^8^ nm/M, 7.383 × 10^−11^ M and 4.16 × 10^−10^ M as the sensitivity, LOD and LOQ respectively. The performance parameters of sensors reported using different methods for AA detection have been compared in Table 2.

#### 6.3.7. Profenofos

**Figure 15:**
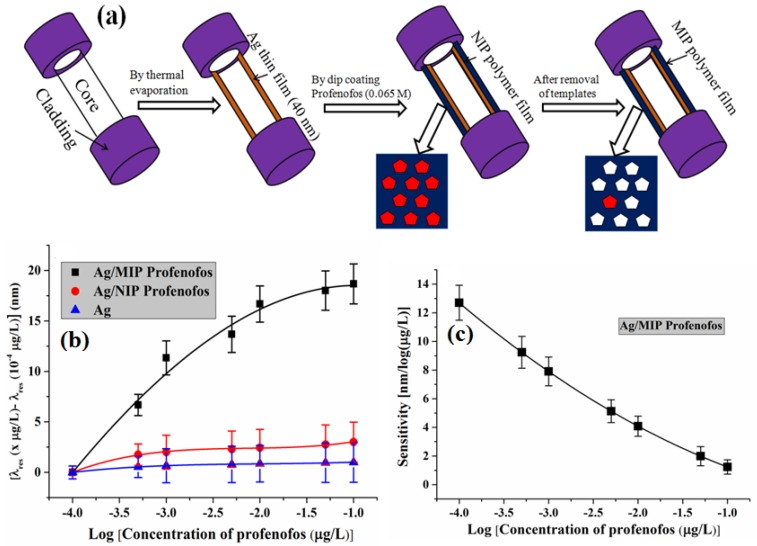
(**a**) The step by step fabrication of FO-SPR-MIP based sensor probe; (**b**) calibration curve and (**c**) sensitivity variation for profenofos concentration in the range 10^−4^–10^−1^ µg/L. Reprinted with permission from [144]. Copyright 2016 Elsevier.

Profenofos is the most common member of the organophosphorus pesticides (OPPs) and is used to control a broad range of pests on cotton, rice, vegetables, sugarcane, etc. OPPs are injurious to human health because OPPs affect the nervous system at very low concentrations [177]. This reason has motivated the development of a sensing method for profenofos detection with high sensitivity and selectivity and low limit of detection. Recently, Shrivastav et al. reported a profenofos detection method using FO-SPR and MIT [144]. The method for the sensing probe fabrication is shown in Figure 15a. The synthesis of MIP was performed by mixing 0.065 mM profenofos (cross-linker), 0.670 mM MAA (monomer) and 0.431 mM TRIM (initiator) in 10 mL DMSO (porogen) in an ultrasonic bath for 10 min and the solution was bubbled under a nitrogen gas environment for 15 min to remove the oxygen from the solution. The solution was then immediately left for pre-polymerization. The unbinding of the profenofos was done by dipping the non-imprinted polymer (NIP)-coated probe in methanol and acetic acid solution 9:1 (v/v) for 10 min. The sensor was calibrated for profenofos solution prepared in aqueous medium with concentration range from 10^−4^ to 10^−1^ µg/L. A red shift of 18.7 nm was observed for this profenofos concentration change. The variations in resonance wavelength shift and the sensitivity of the probe with the concentration of profenofos are shown in Figure 15b,c, respectively. The sensing method shows the lowest detection limit of 2.5 × 10^−6^ µg/L as compared to previously reported studies on profenofos sensing. The sensor’s application for the determination of profenofos in water samples was also tested and it was found that the sensing method shows significant reliability in testing drinking water and tap water samples.

#### 6.3.8. Atrazine

**Figure 16:**
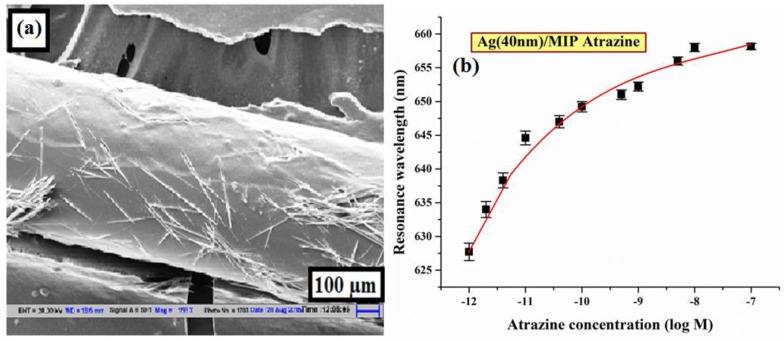
(**a**) SEM micrograph of a MIP-based atrazine sensor showing polymer coated over fiber probe and (**b**) the calibration curve of the sensor for 10^−^^12^–10^−^^7^ M of aqueous atrazine samples. Reprinted with permission from [47]. Copyright 2016 Elsevier.

Atrazine is the member of the triazine class (herbicides), which controls broadleaf weeds in crops, roadway grasses and forestry products, etc. [178]. It belongs to the ‘restricted use pesticide’ (RUP) category, which means that only registered professionals are eligible to use this chemical and its application is strictly restricted for the public. Usually it is found in wastewater samples and due to its toxic nature, it can affect the ecosystem and the human health by several diseases like cancer, reproductive abnormalities, etc., which make its sensing important. A detection method for atrazine sensing was proposed by Agrawal et al. using MIT and the SPR phenomenon over an optical fiber substrate [47]. The sensing probe was fabricated by the deposition of 40 nm thick Ag layer over 1 cm long unclad core of the optical fiber. Further, the MIP layer was coated over the Ag layer. The MIP layer was prepared using two functional monomers MAA and HEMA, while atrazine was used as the template molecule. The operating range of the sensor was kept within the atrazine concentration range of 10^−12^ to 10^−7^ M. The morphological image of polymer coated region over the Ag/core of the fiber is shown in Figure 16a. The sensor possessed the highest sensitivity of 17.34 nm/log M near atrazine concentration of 10^−12^ M. Its limit of detection and limit of quantification reported are 1.92 × 10^−14^ M and 7.61 × 10^−14^ M, respectively. Figure 16b shows the SPR response of the sensor as a function of atrazine concentration. The sensitivity of the sensor was also improved by including a 10 nm thick Al layer between Ag and MIP which resulted in 27% enhancement from the Ag/MIP probe. The highest sensitivity was reported as 22 nm/log M for 10^−12^ atrazine concentration.

#### 6.3.9 Erythromycin

**Figure 17:**
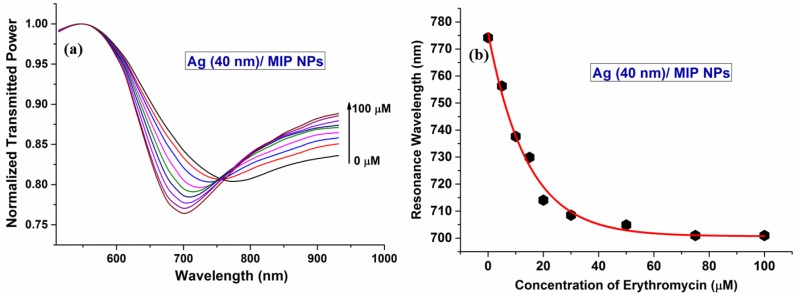
(**a**) SPR spectra and (**b**) variation in resonance wavelength with changing erythromycin concentration from 0–100 µm for SPR and molecularly imprinted nanoparticle-based ERY sensor. Reprinted with permission from [179]. Copyright 2016 The Optical Society (OSA).

Erythromycin (ERY) is a medicine which restricts the activities of Gram positive and Gram negative bacteria, hence is used for the treatment/prevention of bacterial diseases such as skin infections, coughs, etc., but its wide use results in its residues being present in foodstuffs and their derivatives which makes its sensing important. An approach for the detection of ERY in aqueous medium was reported incorporating nanotechnology for the synthesis of MIPs [179]. Nanotechnology was introduced by preparing the ERY imprinted nanoparticles to use as the sensing medium for ERY detection. These were prepared by a two-phase mini-emulsion method [93]. Nanoparticles having ERY as template were coated over the Ag coated fiber core using a dip coating method. The sensor was characterized for the ERY concentration range from 0 to 50 µM. Figure 17a,b show the SPR response and variation in resonance wavelength as a function of ERY sample concentration. The total shift in resonance wavelength was found to be around 72 nm while the sensor showed a detection limit of 5.32 nm/μM.

## 7. Summary and Outlook

In summary, we have discussed fiber optic sensors based on surface plamon resonance (SPR) and localized surface plasmon resonance (LSPR) utilizing the molecular imprinting technique (MIT). The fundamentals of SPR, LSPR and MIT and their implementation for the fabrication of optical fiber sensors have been presented. Further, an overview of the different molecular imprinted polymers, their synthesis methods and basic elements used for MIPs fabrication has been given. The sensors for biological and chemical analytes utilizing SPR/LSPR and MIT techniques and reported in the literature like TNT, vitamin B_2_ and B_3_, tetracycline, melamine, ascorbic acid, profenofos have been discussed along with the performance parameters. A summary of the performance parameters of these fiber optic sensors utilizing SPR/LSPR and MIP techniques is given in Table 3. The advantages of these sensors are that they can be used for the real time monitoring, label free sensing and cost-effectively. The use of the SPR technique improves the sensitivity and response time while the molecular imprinting makes the approach highly specific/selective for the analyte to be detected. Further, SPR/LSPR phenomenon using optical fiber configuration is more beneficial due to the additive advantage of immunity to external EM disturbance and ease of fabrication process for clinical applications. Implementation of optical fiber makes the sensing method capable of in vivo applications for biomedical diagnostics.

The research on SPR/LSPR based fiber optic sensors using molecular imprinting technique has been initiated recently and only a few research papers have been published so far using this tailoring approach. Although, the sensors have various advantages as discussed above, a few parameters like sensitivity, selectivity and the detection limit need further improvement by using MIP nanostructures instead of thin films [179]. The fabrication of MIP polymers using suitable elements and the optimized amount of these is also a big challenge. However, the LSPR/SPR phenomenon can also be made more sensitive using the optimized/mono-dispersed size of metal nanoparticles and layers of metal/metal nanoparticles with not only uniform but also with optimized thicknesses of the layers. The main objective of this article is to show that this research area is wide open and lots of efforts are yet to be made to achieve highly sensitive and selective, quick, and low cost sensors suitable for in vivo applications, ability for real time detection, online monitoring and remote sensing.

## Figures and Tables

**Table 1:** Characterization methods and the information sought for the MIP formation confirmation.

Characterization Technique	Application
SEM, TEM, AFM	Morphological study
FTIR, ATR-FTIR, UV-Vis, NMR	Bonding characteristics, monomer:template screening
XPS, XRD	Structural evaluation
VSM	Magnetic nature analysis
TGA	Thermal analysis

**Table 2:** Linearity and detection limits of various AA detection methods: Reprinted with permission from [66]. Copyright 2016 Institute of Physics.

Method	Substrate/Sensing Layer	Linearity (M)	LOD (M)	Ref.
Electrochemical	DMF exfoliated grapheme	4 × 10^−4^–6 × 10^−3^	1.2 × 10^−4^	[167]
Electrochemical	MIP polypyrrole/pencil graphite electrode	2.7 × 10^−4^–7 × 10^−3^	7.4 × 10^−5^	[168]
Electrochemical	MIP PANI/ITO	5 × 10^−5^–4 × 10^−4^	1.8 × 10^−5^	[169]
Electrochemical	Nitrogen doped porous carbon nanopolyhedra	8 × 10^−5^–2 × 10^−3^	7.4 × 10^−7^	[170]
Electrochemical	Ferricyanide/calcium carbonate microsphere	1 × 10^−6^–2.1 × 10^−3^	7 × 10^−7^	[171]
Colorimetric	Ag Nps/artificial neutral network	2 × 10^−6^–4.8 × 10^−5^	6.2 × 10^−7^	[172]
Electrochemical	Reduced graphene oxide-ZnS nanocomposite	5 × 10^−5^–1 × 10^−3^	3 × 10^−7^	[173]
Electrochemical	Graphene-MWCNT nanocomposite/Au nanoclusters	1 × 10^−5^–1.5 × 10^−4^	2.7 × 10^−7^	[174]
Fluorescence	Protein modified Au nanoclusters	1.5 × 10^−6^–1 × 10^−5^	2 × 10^−7^	[175]
Colorimetric	Pholocatalytic Ag Nps	2.5 × 10^−7^–5 × 10^−5^	7.92 × 10^−10^	[176]
Optical (SPR)	Fiber optic core/Ag/PANI MIP	1 × 10^−8^–1 × 10^−7^	1.28 × 10^−10^	[166]
Optical (LSPR)	Fiber optic core/PANI-Ag MIP	1 × 10^−8^–1 × 10^−6^	1.12 × 10^−10^	[66]
Optical (LSPR + SPR)	Fiber optic core/Ag/PANI-Ag MIP	1 × 10^−8^–1 × 10^−6^	7.38 × 10^−11^	[66]

**Table 3:** Performance parameters of various SPR/LSPR and MIP techniques based fiber optic sensors.

Analyte	Functional Monomer	Fiber Configuration	Operating Range (M)	Sensitivity (nm/M)	LOD (M)	Ref.
TNT	MAA	Flat core/Au/MIP	0–2.5 × 10^−4^	2.7 × 10^4^	5.1 × 10^−5^	[152]
	MAA	Flat core/Au nanostar + MIP	0–2.5 × 10^−4^	8.4 × 10^4^	2.4 × 10^−6^	[153]
TC	AM	Flat core/Ag/MIP hydrogel	0–9.6 × 10^−7^	4.21 × 10^8^	-	[157]
	AM	Flat core/Ag/Ag Np/MIP hydrogel	10^−8^–10^−5^	1.5 × 10^8^	2.2 × 10^−9^	[65]
OTC	AM	Flat core/Ag/MIP hydrogel	0–9.6 × 10^−7^	1.12 × 10^8^	-	[157]
Vitamin B_3_	AM	Flat core/Ag/CCr-MIP	0–0.081	182.557	-	[159]
Vitamin B_2_	AM	Flat core/Ag/CCr-MIP	0–8.5 × 10^−4^	1.28 × 10^4^		[160]
l-Nicotine	MAA	Tapered core/Au/MIP	0–10^−3^	1.3 × 10^4^	1.86 × 10^−4^	[161]
Melamine	MAA	Flat core/Ag/MIP	10^−7^–10^−1^	10.1 × 10^7^	9.87 × 10^−9^	[164]
	MAA	Flat core/Ag/Si/MIP	10^−8^–10^−1^	76.0 × 10^8^	4.3 × 10^−11^	[165]
Ascorbic acid	Aniline	Flat core/Ag/PANI-MIP	10^−8^–10^−4^	26.384 × 10^8^	1.28 × 10^−10^	[166]
	Aniline	Flat core/Ag/PANI-Ag MIP	10^−8^–10^−4^	45.1 × 10^8^	7.38 × 10^−11^	[66]
Profenofos	MAA	Flat core/Ag/MIP	2.68 × (10^−7^–10^−4^)	4.75 × 10^7^	6.69 × 10^−9^	[144]
Atrazine	MAA/HEMA	Flat core/Ag/MIP	10^−12^–10^−7^	17.34 × 10^12^	1.92 × 10^−14^	[47]
ERY	MAA	Flat core/Ag/MIP Nps	0–5 × 10^−5^	5.32 × 10^6^	6.2 × 10^−8^	[179]

The following are the abbreviations and expansions used in Table 3: TNT—trinitrotoluene, MAA—methacrylic acid, TC—tetracycline, OTC—oxytetracycline, AM—acrylamide, CCr—colloidal crystal, PANI—polyaniline, HEMA—2-hydroxyl methacrylate, ERY—erythromycin.

## References

[1] Homola J. (2006). Surface Plasmon Resonance Based Sensors.

[2] Gupta B.D. (2006). Fiber Optic Sensors: Principles and Applications.

[3] Getia S., Gajjar R., Trivedi P. (2013). Classification of fiber optical sensors. Int. J. Electron. Commun. Comput. Technol..

[4] Gupta B.D., Srivastava S.K., Verma R. (2015). Fiber Optic Sensors Based on Plasmonics.

[5] Lopez-Higuera J.M. (2002). Handbook of Optical Fiber Sensor Technology.

[6] Geddes C.D. (2011). Reviews in Plasmonics 2010.

[7] Yin S., Ruffin P.B., Yu F.T.S. (2008). Fiber Optic Sensors, Second Edition.

[8] Bhatia P., Gupta B.D. (2013). Surface plasmon resonance based fiber optic refractive index sensor utilizing silicon layer: Effect of doping. Opt. Commun..

[9] Singh S., Mishra S.K., Gupta B.D. (2013). Sensitivity enhancement of a surface plasmon resonance based fiber optic refractive index sensor utilizing an additional layer of oxides. Sens. Actuators A Phys..

[10] Mishra S.K., Varshney C., Gupta B.D. Sensitivity enhancement of surface plasmon resonance based fiber optic refractive index sensor using an additional layer of zinc oxide. Proceedings of the Fifth European Workshop on Optical Fibre Sensors.

[11] Whitcombe M.J., Kirsch N., Nicholls I.A. (2014). Molecular imprinting science and technology: A survey of the literature for the years 2004–2011. J. Mol. Recognit..

[12] Lofgreen J.E., Ozin G.A. (2014). Controlling morphology and porosity to improve performance of molecularly imprinted sol-gel silica. Chem. Soc. Rev..

[13] Alexander C., Andersson H.S., Andersson L.I., Ansell R.J., Kirsch N., Nicholls I.A., O’Mahony J., Whitcombe M.J. (2006). Molecular imprinting science and technology: A survey of the literature for the years up to and including 2003. J. Mol. Recognit..

[14] Polyakov M., Khim Z. (1931). Adsorption properties and structure of silica gel. Zh. Fiz. Khim. Ser. B.

[15] Chen L., Xu S., Li J. (2011). Recent advances in molecular imprinting technology: Current status, challenges and highlighted applications. Chem. Soc. Rev..

[16] Xu S., Lu H., Zheng X., Chen L. (2013). Stimuli-responsive molecularly imprinted polymers: Versatile functional materials. J. Mater. Chem. C.

[17] Schirhagl R. (2013). Bioapplications for molecularly imprinted polymers. Anal. Chem..

[18] Castell O.K., Barrow D.A., Kamarudin A.R., Allender C.J. (2011). Current practices for describing the performance of molecularly imprinted polymers can be misleading and may be hampering the development of the field. J. Mol. Recognit..

[19] Hoshino Y., Shea K.J. (2011). The evolution of plastic antibodies. J. Mater. Chem..

[20] Li S., Cao S., Whitcombe M.J., Piletsky S.A. (2014). Size matters: Challenges in imprinting macromolecules. Prog. Polym. Sci..

[21] Xu Z., Uddin K.M.A., Kamra T., Schnadt J., Ye L. (2014). Fluorescent boronic acid polymer grafted on silica particles for affinity separation of saccharides. ACS Appl. Mater. Interfaces.

[22] Nicholls I.A., Karlsson B.R.C., Olsson G.D., Rosengren A.M. (2013). Computational strategies for the design and study of molecularly imprinted materials. Ind. Eng. Chem. Res..

[23] Han Y., Yuan X., Zhu M., Li S., Whitcombe M.J., Piletsky S.A. (2014). A catalytic and shape-memory polymer reactor. Adv. Funct. Mater..

[24] Qiu H., Fan L., Li X., Li L., Sun M., Luo C. (2013). A microflow chemiluminescence sensor for indirect determination of dibutyl phthalate by hydrolyzing based on biological recognition materials. J. Pharm. Biomed. Anal..

[25] Komiyama M., Takeuchi T., Mukawa T., Asanuma H. (2003). Molecular Imprinting from Fundamentals to Applications.

[26] Ye L. (2013). Molecular Imprinting: Principles and Applications of Micro- and Nanostructure Polymers.

[27] Lee S.W., Kunitake T. (2012). Handbook of Molecular Imprinting: Advanced Sensor Applications.

[28] Kretchmann E., Reather E. (1968). Radiative decay of nonradiative surface plasmons excited by light. Z. Naturforsch. A Phys. Sci..

[29] Olaru A., Bala C., Jaffrezic-Renault N., Aboul-Enein H.Y. (2015). Surface plasmon resonance (SPR) biosensors in pharmaceutical analysis. Crit. Rev. Anal. Chem..

[30] Couture M., Zhao S.S., Masson J. (2013). Modern surface plasmon resonance for bioanalytics and biophysics. Phys. Chem. Chem. Phys..

[31] Sharma A.K., Jha R., Gupta B.D. (2007). Fiber-optic sensors based on surface plasmon resonance: A comprehensive review. IEEE. Sens. J..

[32] Gupta B.D., Verma R.K. (2009). Surface plasmon resonance-based fiber optic sensors: Principle, probe designs, and some applications. J. Sens..

[33] Fakonas J.S., Mitskovets A., Atwater H.A. (2105). Path entanglement of surface plasmons. New J. Phys..

[34] Dionne J.A., Sweatlock L.A., Sheldon M.T., Alivisatos A.P., Atwater H.A. (2010). Silicon based plasmonics for on-chip photonics. IEEE J. Selec. Top. Quant. Electron..

[35] Maier S.A. (2007). Plasmonics: Fundamentals and Applications.

[36] Homola J. (2003). Present and future of surface plasmon resonance biosensors. Anal. Bioanal. Chem..

[37] Liedberg B., Nylander C., Lunstrom I. (1983). Surface plasmon resonance for gas detection and biosensing. Sens. Actuators.

[38] Arghir I., Delport F., Spasic D., Lammertyn J. (2015). Smart design of fiber optic surfaces for improved plasmonic biosensing. New Biotechnol..

[39] Villuendas F., Pelayo J. (1990). Optical fiber device for chemical sensing based on surface plasmon excitridon. Sens. Actuators A Phys..

[40] Jorgenson R.C., Yee S.S. (1993). A fiber-optic chemical sensor based on surface plasmon resonance. Sens. Actuators B Chem..

[41] Slavik R., Homola J., Ctyroky J., Brynda E. (2001). Novel spectral fiber optic sensor based on surface plasmon resonance. Sens. Actuators B Chem..

[42] Harris R.D., Wilkinson J.S. (1995). Waveguide surface plasmon resonance sensors. Sens. Actuators B Chem..

[43] Gupta B.D., Sharma A.K. (2005). Sensitivity evaluation of a multi-layered surface plasmon resonance-based fiber optic sensor: A theoretical study. Sens. Actuators B Chem..

[44] Markatos S., Zervas M.N., Giles I.P. (1988). Optical fiber surface plasmon wave devices. Electron. Lett..

[45] Hu J., Sun X., Agarwal A., Kimerling L.C. (2009). Design guidelines for optical resonator biochemical sensors. J. Opt. Soc. Am. B.

[46] White I.M., Fan X. (2008). On the performance quantification of resonant refractive index sensors. Opt. Exp..

[47] Agrawal H., Shrivastav A.M., Gupta B.D. (2016). Surface plasmon resonance based optical fiber sensor for atrazine detection using molecular imprinting technique. Sens. Actuators B Chem..

[48] Srivastava S.K., Gupta B.D. (2011). Multi-tapered fiber optic SPR Sensor with enhanced sensitivity. IEEE Photonics Technol. Lett..

[49] Kim Y.C., Peng W., Banerji S., Booksh K.S. (2005). Tapered fiber optic surface plasmon resonance sensor for analyses of vapour and liquid phases. Opt. Lett..

[50] Verma R.K., Sharma A.K., Gupta B.D. (2008). Surface plasmon resonance based tapered fiber optic sensor with different taper profiles. Opt. Commun..

[51] Monzon-Hernandez D., Villatoro J., Talavera D., Luna-Moreno D. (2004). Optical-fiber surface-plasmon resonance sensor with multiple resonance peaks. Appl. Opt..

[52] Verma R.K., Gupta B.D. (2008). Theoretical modelling of a bidimensional U-shaped surface plasmon resonance based fibre optic sensor for sensitivity enhancement. J. Phys. D Appl. Phys..

[53] Srivastava S.K., Arora V., Sapra S., Gupta B.D. (2012). Localized surface plasmon resonance based fiber optic U-shaped biosensor for the detection of blood glucose. Plasmonics.

[54] Wang S.F., Chiu M.H., Hsu J.C., Chang R.S., Wang F.T. (2005). Theoretical analysis and experimental evaluation of D-type optical fiber sensor with a thin gold film. Opt. Commun..

[55] Sharma A.K., Gupta B.D. (2005). On the sensitivity and signal to noise ratio of a step-index fiber optic surface plasmon resonance sensor with bimetallic layers. Opt. Commun..

[56] Tabassum R., Gupta B.D. (2016). SPR based fiber-optic sensor with enhanced electric field intensity and figure of merit using single and bimetallic configurations. Opt. Commun..

[57] Lahav A., Auslender M., Abdulhalim I. (2008). Sensitivity enhancement of guided-wave surface-plasmon resonance sensors. Opt. Lett..

[58] Shalabney A., Abdulhalim I. (2010). Electromagnetic fields distribution in multilayer thin film structures and the origin of sensitivity enhancement in surface plasmon resonance sensors. Sens. Actuators B Chem..

[59] Petryayeva E., Krull U.J. (2011). Localized surface plasmon resonance: Nnostructures, bioassays and biosensing-A review. Anal. Chim. Acta.

[60] Mayer K.M., Hafner J.H. (2011). Localized surface plasmonn resonance sensors. Chem. Rev..

[61] Srivastava S.K., Verma R.K., Gupta B.D. (2009). Theoretical modeling of a localized surface plasmon resonance based intensity modulated fiber optic refractive index sensor. Appl. Opt..

[62] Guo L., Jackman J.A., Yang H., Chen P., Cho N., Kim D. (2015). Strategies for enhancing the sensitivity of plasmonic nanosensors. Nano Today.

[63] Dutta R., Singh B.P., Kundu T. (2013). Plasmonic coupling effect on spectral response of silver nanoparticles immobilized on an optical fiber sensor. J. Phy. Chem. C.

[64] Garcia M.A. (2012). Corrigendum: Surface plasmon in metallic nanoparticles: Fundementals and applications. J. Phys. D Appl. Phys..

[65] Shrivastav A.M., Mishra S.K., Gupta B.D. (2015). Localized and propagating surface plasmon resonance based fiber optic sensor for the detection of tetracycline using molecular imprinting. Mat. Res. Exp..

[66] Shrivastav A.M., Usha S.P., Gupta B.D. (2016). Localized and propagating SPR and molecular imprinting based fiber optic ascorbic acid sensor using in-situ polymerized polyaniline-Ag nanocomposite. Nanotechnol..

[67] Chen L., Wang X., Lu W., Wu X., Li J. (2016). Molecular imprinting: Perspectives and applications. Chem. Soc. Rev..

[68] Yan H., Row K.H. (2006). Characteristic and synthetic approach of molecularly imprinted polymer. Int. J. Mol. Sci..

[69] Kryscio D.R., Peppas N.A. (2012). Critical review and perspective of macromolecularly imprinted polymers. Acta Biomater..

[70] Shea K.J., Sasaki D.Y. (1991). An analysis of small-molecule binding to functionalized synthetic polymers by 13C CP/MAS NMR and FT-IR spectroscopy. J. Am. Chem. Soc..

[71] Wulff G., Vesper W., Grobe-Einsler R., Sarhan A. (1977). Enzyme-analogue built polymers, 4. On the synthesis of polymers containing chiral cavities and their use for the resolution of acemates. Makromol. Chem..

[72] Verheyen E., Schillemans J.P., van Wijk M., Demeniex M.A., Hennink W.E., van Nostrum C.F. (2011). Challenges for the effective molecular imprinting of proteins. Biomaterials.

[73] Molecular Imprinted Polymers: A Review. http://shodhganga.inflibnet.ac.in/bitstream/10603/7082/13/13_chapter%202.pdf.

[74] Vlatakis G., Andersson L.I., Muller R., Mosbach K. (1993). Drug assay using antibody mimics made by molecular imprinting. Nature.

[75] Arshady R., Mosbach K. (1981). Synthesis of substrate-selective polymers by host-guest polymerization. Macromol. Chem..

[76] Umpley R.J., Baxter S.C., Chen Y., Shah R.N., Shimizu K.D. (2001). Characterization of molecularly imprinted polymers with the Langmuir-Freundlich isotherm. Anal Chem..

[77] Cormack P.A.G., Elorza A.Z. (2004). Molecularly imprinted polymers: Synthesis and characterisation. J. Chromatogr. B.

[78] Mosbach K., Ramson O. (1996). The emerging technique of molecular imprinting and its future impact on biotechnology. Biotechnology.

[79] Mayes A.G., Mosbach K. (1996). Molecularly imprinted polymer beads: Suspension polymerization using a liquid perfluorocarbon as the dispersing phase. Anal. Chem..

[80] Peng H., Liang C., Zhou A., Zhang Y., Xie Q., Yao S. (2000). Development of a new atropine sulfate bulk acoustic wave sensor based on a molecularly imprinted electrosynthesized copolymer of aniline with o-phenylenediamine. Anal. Chim. Acta.

[81] Wulff G. (1995). Molecular imprinting in cross-linked materials with the aid of molecular templates- A way towards artificial antibodies. Angew. Chem. Int. Ed. Engl..

[82] Hosoya K., Yoshihako K., Shirasu Y., Kimata K., Araki T., Tanaka N., Haginaka J. (1996). Molecularly imprinted uniform-size polymer-based stationary phase for high-performance liquid chromatography structural contribution of cross-linked polymer network on specific molecular recognition. J. Chromatogr. A.

[83] Wang B., Wang Y., Yang H., Wang J., Deng A. (2011). Preparation and characterization of molecularly imprinted microspheres for selective extraction of trace melamine from milk samples. Microchim. Acta.

[84] Dvorakova G., Haschick R., Chiad K., Klapper M., Mullen K., Biffis A. (2010). Molecularly imprinted nanospheres by nonaqueous emulsion polymerization. Macromol. Rapid Commun..

[85] Hiratsuka Y., Funaya N., Matsunaga H., Haginaka J. (2013). Preparation of magnetic molecularly imprinted polymers for bisphenol A and its analogues and their application to the assay of bisphenol A in river water. J. Pharm. Biomed. Anal..

[86] Wang X., Mao H., Huang W., Guan W., Zou X., Pan J., Yan Y. (2011). Preparation of magnetic imprinted polymer particles via microwave heating initiated polymerization for selective enrichment of 2-amino-4-nitrophenol from aqueous solution. Chem. Eng. J..

[87] Liu Y., Hoshina K., Haginaka J. (2010). Monodispersed, molecularly imprinted polymers for cinchonidine by precipitation polymerization. Talanta.

[88] Alizadeh T. (2010). Preparation of molecularly imprinted polymer containing selective cavities for urea molecule and its application for urea extraction. Anal. Chim. Acta.

[89] Ramstroem O., Andersson L.I., Mosbach K. (1993). Recognition sites incorporating both pyridinyl and carboxy functionalities prepared by molecular imprinting. J. Org. Chem..

[90] Zhang Z., Li M., Ren J., Qu X. (2015). Cell-imprinted antimicrobial bionanomaterials with tolerable toxic side effects. Small.

[91] Hayden O., Lieberzeit P.A., Blaas D., Dickert F.L. (2006). Artificial antibodies for bioanalyte detection-sensing viruses and proteins. Adv. Funct. Mater..

[92] Hayden O., Mann K.J., Krassnig S., Dickert F.L. (2006). Biomimetic ABO blood-group typing. Angew. Chem. Int. Ed..

[93] Wackerlig J., Lieberzeit P.A. (2015). Molecularly imprinted polymer in nanoparticles in chemical sensing-synthesis, characterization and application. Sens. Actuators B Chem..

[94] Gao D., Zhang Z., Wu M., Xie C., Guan G., Wang D. (2007). A surface functional monomer-directing strategy for highly dense imprinting of TNT at surface of silica nanoparticles. J. Am. Chem. Soc..

[95] Xie C., Liu B., Wang Z., Gao D., Guan G., Zhang Z. (2008). Molecular imprinting at walls of silica nanotubes for TNT recognition. Anal. Chem..

[96] Li Y., Yang H.H., You Q.H., Zhuang Z.X., Wang X.R. (2006). Protein recognition via surface molecularly imprinted polymer nanowires. Anal. Chem..

[97] Sreenivasan K., Sivakumar R. (1999). Imparting recognition sites in poly(HEMA) for two compounds through molecular imprinting. J. Appl. Polym. Sci..

[98] Haruki M., Konnai Y., Shimada A., Takeuchi H. (2007). Molecularly imprinted polymer-assisted refolding of lysozyme. Biotechnol. Prog..

[99] Rachkov A., Minoura N. (2000). Recognition of oxytocin and oxytocin-related peptides in aqueous media using a molecularly imprinted polymer synthesized by the epitope approach. J. Chromatogr. A.

[100] Kareuhanon W., Lee V.S., Nimmanpipug P., Tayapiwatana C., Pattarawarapan M. (2009). Synthesis of molecularly imprinted polymers for nevirapine by dummy template imprinting approach. Chromatographia.

[101] Takano E., Taguchi Y., Ooya T., Takeuchi T. (2012). Dummy template-imprinted polymers for bisphenol A prepared using a schiff base-type template molecule with post-imprinting oxidation. Anal. Lett..

[102] Zhang Z., Li J., Song X., Ma J., Chen L. (2014). Hg^2+^ ion-imprinted polymers sorbents based on dithizone–Hg^2+^ chelation for mercury speciation analysis in environmental and biological samples. RSC Adv..

[103] Jakusch M., Janota M., Mizaikoff B., Mosbach K., Haupt K. (1999). Molecularly imprinted polymers and infrared evanescent wave spectroscopy. A chemical sensors approach. Anal. Chem..

[104] Sergeyeva T.A., Piletsky S.A., Panasyuk T.L., Elskaya A.V., Brovko A.A., Slinchenko E.A., Sergeeva L.M. (1999). Conductimetric sensor for atrazine detection based on molecularly imprinted polymer membranes. Analyst.

[105] Baggiani C., Trotta F., Giraudi G., Giovannoli C., Vanni A. (1999). A molecularly imprinted polymer for the pesticide bentazone. Anal. Commun..

[106] Cai D., Ren L., Zhao H., Xu C., Zhang L., Yu Y., Wang H., Lan Y., Roberts M.H., Chuang J.H. (2010). A molecular imprint nanosensor for ultrasensitive detection of proteins. Nat. Nanotechnol..

[107] Zhang W., He X.W., Chen Y., Li W.Y., Zhang Y.K. (2011). Composite of CdTe quantum dots and molecularly imprinted polymer as a sensing material for cytochrome c. Biosens. Bioelectron..

[108] Golker K., Karlsson B.R.C., Olsson G.D., Rosengren A.M., Nicholls I.A. (2013). Influence of composition and morphology on template recognition in molecularly imprinted polymers. Macromolecules.

[109] Li P., Wang T., Lei F., Tang P., Tan X., Liu Z., Shen L. (2014). Rosin-based molecularly imprinted polymers as the stationary phase in high-performance liquid chromatography for selective separation of berberine hydrochloride. Polym. Int..

[110] Saloni J., Lipkowski P., Dasary S.S.R., Anjaneyulu Y., Yu H., Hill G. (2011). Theoretical study of molecular interactions of TNT, acrylic acid, and ethylene glycol dimethacrylate—Elements of molecularly imprinted polymer modeling process. Polymer.

[111] Booker K., Holdsworth C.I., Doherty C.M., Hill A.J., Bowyer M.C., McCluskey A. (2014). Ionic liquids as porogens for molecularly imprinted polymers: Propranolol, a model study. Org. Biomol. Chem..

[112] Wulff G., Kemmerer R., Vietmeier J., Poll H.G. (1982). The preparation of chiral cavities in synthetic polymers. Nouv. J. Chim..

[113] Shea K.J., Thompson E.A. (1978). Template synthesis of macromolecules. Selective functionalization of an organic polymer. J. Org. Chem..

[114] Saloni J., Walker K., Hill G. (2013). Theoretical investigation on monomer and solvent selection for molecular imprinting of nitrocompounds. J. Phys. Chem. A.

[115] He J., Lv R., Cheng J., Li Y., Xue J., Lu K., Wang F. (2010). Preparation and characterization of molecularly imprinted microspheres for dibutyl phthalate recognition in aqueous environment. J. Sep. Sci..

[116] Masque N., Marce R.M., Borrul F. (2001). Molecularly imprinted polymers: New tailor-made materials for selective solid-phase extraction. TrAC Trends Anal. Chem..

[117] O’Shannessy D.J., Ekberg B., Mosbach K. (1989). Molecular imprinting of amino acid derivatives at low temperature (0 °C) using photolytic homolysis of azobisnitrile. Anal. Biochem..

[118] Sellergren B. (1989). Molecular imprinting by noncovalent interactions-enantioselectivity and binding-capacity of polymers prepared under conditions favoring the formation of template complexes. Macromol. Chem..

[119] Gallego-Gallegos M., Munaz-Olivas R., Camara C., Mancheno M.J., Sierra M.A. (2006). Synthesis of a pH dependent covalent imprinted polymer able to recognize organotin species. Analyst.

[120] Sellergren B. (1994). Direct drug determination by selective sample enrichment on an imprinted polymer. Anal. Chem..

[121] Muldoon M.T., Stanker L.H. (1997). Molecularly imprinted solid phase extraction of atrazine from beef liver extracts. Anal. Chem..

[122] Zander A., Findlay P., Renner T., Sellergren B., Swietlow A. (1998). Analysis of nicotine and its oxidation products in nicotine chewing gum by a molecularly imprinted solid-phase extraction. Anal. Chem..

[123] Sekine S., Watanabe Y., Yoshimi Y., Hattori K., Sakai K. (2007). Influence of solvents on chiral discriminative gate effect of molecularly imprinted poly(ethylene glycol dimethacrylate-co-methacrylic acid). Sens. Actuators B Chem..

[124] Andersson L.I., Muller R., Vlatakis G., Mobach K. (1995). Mimics of the binding sites of opioid receptors obtained by molecular imprinting of enkephalin and morphine. Proc. Natl. Acad. Sci. USA.

[125] Mayes A.G., Andersson L.I., Mosbach K. (1994). Sugar binding polymers showing high anomeric and epimeric discrimination obtained by noncovalent molecular imprinting. Anal. Biochem..

[126] Andersson L.I. (1996). Application of molecular imprinting to the development of aqueous buffer and organic solvent based radioligand binding assays for (s)-propranolol. Anal. Chem..

[127] Hedborg E., Winquist F., Lundstrom I., Andersson L.I., Mosbach K. (1993). Some studies of molecularly-imprinted polymer membranes in combination with field-effect devices. Sens. Actuators A Phys..

[128] Piletsky S.A., Piletska E.V., Elgersma A.V., Yano K., Parhometz Y.P., El’skaya A.V., Karube I. (1995). Atrazine sensing by molecularly imprinted membranes. Biosens. Bioelectron..

[129] Kriz D., Mosbach K. (1995). Competitive amperometric morphine sensor based on an agarose immobilised molecularly imprinted polymer. Anal. Chim. Acta.

[130] Tiwari M.P., Prasad A. (2015). Molecularly imprinted polymer based enantioselective sensing devices: A review. Anal. Chim. Acta.

[131] Andersson L.I., Miyabayashi A., Oshannessy D.J., Mosbach K. (1990). Enantiomeric resolution of amino acid derivatives on molecularly imprinted polymers as monitored by potentiometric measurements. J. Chromatogr. A.

[132] Panasyuk T.L., Mirsky V.M., Piletsky S.A., Wolfbeis O.S. (1999). Electropolymerized molecularly imprinted polymers as receptor layers in capacitive chemical sensors. Anal. Chem..

[133] Turkewitsch P., Wandelt B., Darling G.D., Powell W.S. (1998). Fluorescent functional recognition sites through molecular imprinting. A polymer-based fluorescent chemosensor for aqueous cAMP. Anal. Chem..

[134] Descalzo A.B., Somoza C., Bondi M.C.M., Orellana G. (2013). Luminescent core–shell imprinted nanoparticles engineered for targeted förster resonance energy transfer-based sensing. Anal. Chem..

[135] Liu C., Song Z., Pan J., Wei X., Gao L., Yan Y., Li L., Wang J., Chen R., Dai J. (2013). Molecular imprinting in fluorescent particle stabilized pickering emulsion for selective and sensitive optosensing of λ-cyhalothrin. J. Phys. Chem. C.

[136] Yu J., Wan F., Zhang C., Yan M., Zhang X., Wang S. (2010). Molecularly imprinted polymeric microspheres for determination of bovine serum albumin based on flow injection chemiluminescence sensor. Biosens. Bioelectron..

[137] Zhou J., Gan N., Hu F., Li T., Zhou H., Li X., Zheng L. (2013). A single antibody sandwich electrochemiluminescence immunosensor based on protein magnetic molecularly imprinted polymers mimicking capture probes. Sens. Actuators B Chem..

[138] Li S.P., Guan H.M., Xu G.B., Tong Y.J. (2015). Molecular imprinting electrochemiluminescence analysis. Chin. J. Anal. Chem..

[139] Li J., Zhang Z., Xu S., Chen L., Zhou N., Xiong H., Peng H. (2011). Label-free colorimetric detection of trace cholesterol based on molecularly imprinted photonic hydrogels. J. Mater. Chem..

[140] Wu Z., Tao C., Lin C., Shen D., Li G. (2008). Label-free colorimetric detection of trace atrazine in aqueous solution by using molecularly imprinted photonic polymers. Chem. Eur. J..

[141] Sergeyeva T.A., Gorbach L.A., Piletska E.V., Piletsky S.A., Brovko O.O., Honcharova L.A., Lutsyk O.D., Sergeeva L.M., Zinchenko O.A., El’skaya A.V. (2013). Colorimetric test-systems for creatinine detection based on composite molecularly imprinted polymer membranes. Anal. Chim. Acta.

[142] Lai E.P.C., Fafara A., Noot V.A.V., Kono M., Polsky B. (1998). Surface plasmon resonance sensors using molecularly imprinted polymers for sorbent assay of theophylline, caffeine, and xanthine. Can. J. Chem..

[143] Zhao N., Chen C., Zhou J. (2012). Surface plasmon resonance detection of ametryn using a molecularly imprinted sensing film prepared by surface-initiated atom transfer radical polymerization. Sens. Actuators B Chem..

[144] Shrivastav A.M., Usha S.P., Gupta B.D. (2016). Fiber optic profenofos sensor based on surface Plasmon resonance technique and molecular imprinting. Biosens. Bioelectron..

[145] Wang Y., Yan B., Chen L. (2013). SERS tags: Novel optical nanoprobes for bioanalysis. Chem. Rev..

[146] Chang L., Ding Y., Li X. (2013). Surface molecular imprinting onto silver microspheres for surface enhanced raman scattering applications. Biosens. Bioelectron..

[147] Ye J., Chen Y., Liu Z. (2014). A boronate affinity sandwich assay: An appealing alternative to immunoassays for the determination of glycoproteins. Angew. Chem. Int. Ed..

[148] Usha S.P., Shrivastav A.M., Gupta B.D. (2016). FO-SPR based dextrose sensor using Ag/ZnO nanorods/GOx for insulinoma detection. Biosens. Bioelectron..

[149] Semval V., Shrivastav A.M., Verma R., Gupta B.D. (2016). Surface plasmon resonance based fiber optic ethanol sensor using layers of silver/silicon/hydrogel entrapped with ADH/NAD. Sens. Actuators B Chem..

[150] LSPR vs. SPR: What’s the Difference?-Nicoya Lifesciences. https://nicoyalife.com/technology/surface-plasmon-resonance/localized-surface-plasmon-resonance-theory/.

[151] Willets K.A., Duyne R.P.V. (2007). Localized surface plasmon resonance spectroscopy and sensing. Annu. Rev. Phys. Chem..

[152] Cennamo N., D’Agostino G., Galatus R., Bibbò L., Pesavento M., Zeni L. (2013). Sensors based on surface plasmon resonance in a plastic optical fiber for the detection of trinitrotoluene. Sens. Actuators B Chem..

[153] Pesavento M., Cennamo N., Donà A., Pallavicini P., D’Agostino G., Zenuthor L.A. A new approach for selective optical fiber sensors based on localized surface plasmon resonance of gold nanostars in molecularly imprinted polymer. Proceedings of the Recent Advances in Biomedical & Chemical Engineering and Materials Science.

[154] Pallavicini P., Donà A., Casu A., Chirico G., Collini M., Dacarro G., Falqui A., Milanese C., Sironic L., Taglietti A. (2013). Triton X-100 for three Plasmon gold nanostars with two photothermally active NIR (near IR) and SWIR (short-wavelength IR) channels. Chem. Commun..

[155] Masawat P., Slater J.M. (2007). The determination of tetracycline residues in food using a disposable screen-printed gold electrode (SPGE). Sens. Actuators B Chem..

[156] Van den Bogaard A.E., Stobberingh E.E. (2000). Epidemiology of resistance to antibiotics. Links between animals and humans. Int. J. Antimicrob. Agents.

[157] Verma R., Gupta B.D. (2013). Optical fiber sensor for the detection of tetracycline using surface plasmon resonance and molecular imprinting. Analyst.

[158] Zou J., Xu Y., Hou B., Wu D., Sun Y. (2007). Controlled growth of silver nanoparticles in a hydrothermal process. China Part..

[159] Verma R., Gupta B.D. (2013). Fiber optic SPR sensor for the detection of 3-pyridinecarboxamide (Vitamin B_3_) using molecularly imprinted hydrogel. Sens. Actuators B Chem..

[160] Verma R., Gupta B.D. Surface plasmon resonance based optical fiber riboflavin sensor by using molecularly imprinted gel. Proceedings of the Fifth European Workshop on Optical Fibre Sensors.

[161] Cennamo N., D’Agostino G., Pesavento M., Zenia L. (2014). High selectivity and sensitivity sensor based on MIP and SPR in tapered plastic optical fibers for the detection of l-nicotine. Sens. Actuators B Chem..

[162] Tyan Y., Yang M., Jong S., Wang C., Shiea J. (2009). Melamine contamination. Anal. Bioanal. Chem..

[163] Kennaway E.L. (1921). The estimation of non-protein nitrogen in blood by a micro-Kjeldahl method. Biochem. J..

[164] Shrivastav A.M., Mishra S.K., Gupta B.D. (2015). Fiber optic SPR sensor for the detection of melamine using molecular imprinting. Sens. Actuators B Chem..

[165] Shrivastav A.M., Gupta B.D. (2016). SPR and molecular imprinting based fiber optic melamine sensor with high sensitivity and low limit of detection. IEEE J. Sel. Top. Quantum Electron..

[166] Shrivastav A.M., Mishra S.K., Gupta B.D. (2015). Surface plasmon resonance based fiber optic sensor for the detection of ascorbic acid utilizing molecular imprinted polyaniline film. Plasmonics.

[167] Kelly G.P., Neill A.O., McEvoy N., Peltekis N., Coleman J.N., Duesberg G.S. (2010). Electrochemical ascorbic acid sensor based on DMF-exfoliated grapheme. J. Mater. Chem..

[168] Ozcan L., Sahin M., Sahin Y. (2008). Electrochemical preparation of a molecularly imprinted polypyrrole-modified pencil graphite electrode for determination of ascorbic acid. Sensors.

[169] Roy A.K., Nisha V.S., Dhand C., Malhotra B.D. (2011). Molecularly imprinted polyaniline film for ascorbic acid detection. J. Mol. Recog..

[170] Gai P., Zhang H., Zhang Y., Liu W., Zhu G., Zhang X., Chen J. (2013). Simultaneous electrochemical detection of ascorbic acid, dopamine and uric acid based on nitrogen doped porous carbon nanopolyhedra. J. Mater. Chem. B.

[171] Li F., Tang C., Liu S., Ma G. (2010). Development of an electrochemical ascorbic acid sensor based on the incorporation of a ferricyanide mediator with a polyelectrolyte–calcium carbonate microsphere. Electrochim. Acta.

[172] Nezhad M.R.H., Tashkhourian J., Khodaveisi J., Khoshi M.R. (2010). Simultaneous colorimetric determination of dopamine and ascorbic acid based on the surface plasmon resonance band of colloidal silver nanoparticles using artificial neural networks. Anal. Methods.

[173] Yang Y.G. (2015). One-pot synthesis of reduced graphene oxide/zinc sulfide nanocomposite at room temperature for simultaneous determination of ascorbic acid, dopamine and uric acid. Sens. Actuators B Chem..

[174] Abdelwahab A.A., Shim Y.B. (2015). Simultaneous determination of ascorbic acid, dopamine, uric acid and folic acid based on activated graphene/MWCNT nanocomposite loaded Au nanoclusters. Sens. Actuators B Chem..

[175] Wang X., Wu P., Hou X., Lv Y. (2013). An ascorbic acid sensor based on protein-modified Au nanoclusters. Analyst.

[176] Peng J., Ling J., Zhang X.Q., Zhang L.Y., Cao Q.E., Ding Z.T. (2015). A rapid, sensitive and selective colorimetric method for detection of ascorbic acid. Sens. Actuators B Chem..

[177] Guerrieri A., Monaci L., Quinto M., Palmisano F. (2002). A disposable amperometric biosensor for rapid screening of anticholinesterase activity in soil extracts. Analyst.

[178] Shaner D.L., Krutz L.J., Henry W.B., Hanson B.D., Poteet M.D., Rainbolt C.R. (2010). Sugarcane soils exhibit enhanced atrazine degradation and cross adaptation to other s-triazines. J. Am. Soc. Sugar Cane Technol..

[179] Gupta B.D., Shrivastav A.M., Usha S.P. Fiber optic SPR nanosensor for erythromycin detection using molecularly imprinted nanoparticles. Proceedings of the CLEO: Science and Innovations.

